# Supplementary material for: Host control of persistent Epstein–Barr virus infection
Source: Nature. 2026 Feb 19;653(8114):444–54. doi: 10.1038/s41586-026-10274-4 (PMC13171444; doi:10.1038/s41586-026-10274-4)
Supplement: Supplementary file 1 — This file contains Supplementary Notes 1–15, including Supplementary Figs 1–9. [file 41586_2026_10274_MOESM1_ESM.pdf]

nature portfolio

https://doi.org/10.1038/s41586-026-10274-4

Supplementary information

Host control of persistent Epstein–Barr virus infection

In the format provided by the authors and unedited

Host control of persistent Epstein–Barr virus infection – Supplementary Notes

Authors: Axel Schmidt, T. Madhusankha Alawathurage, Friederike S. David, Yosuke Ogawa, Leonard Frach, Sylvia Richter, Merle Schaefer, Carina M. Mathey, Sabrina K. Henne, Japan COVID-19 Task Force, Andreas J. Forstner, Alexander T. Dilthey, Anne-Katrin Pröbstel, Kaan Boztug, Markus M. Nöthen, Ho Namkoong, Yukinori Okada, Eva C. Beins, Kerstin U. Ludwig

Table of Contents

Supplementary Note 1: Study design - Cross-biobank analyses of EBVread+ 2
Supplementary Note 2: Extraction of EBV-reads in UK Biobank data 3
Supplementary Note 3: Additional information for AoU analyses 5
Supplementary Note 4: Analysis of covariates in UK Biobank 6
Supplementary Note 5: Analysis of JCTF data (paired GS/bulk RNA seq) 8
Supplementary Note 6: Extraction of HHV7-reads in UK Biobank data 9
Supplementary Note 7: Monogenic susceptibility for EBV infection 11
Supplementary Note 8: Additional information on Two-sample MR 12
Supplementary Note 9: Generation of genetic variant sets in UK Biobank data 13
Supplementary Note 10: Analysis of MHC region with and without SPA 14
Supplementary Note 11: Epistatic analysis 16
Supplementary Note 12: HLA imputation in AoU 17
Supplementary Note 13: Details on the validation cohorts 19
Supplementary Note 14: Annotation of 27 non-MHC risk loci 21
Supplementary Note 15: scDRS analyses 29
References Supplementary Information 30

Supplementary Note 1: Study design - Cross-biobank analyses of EBVread+

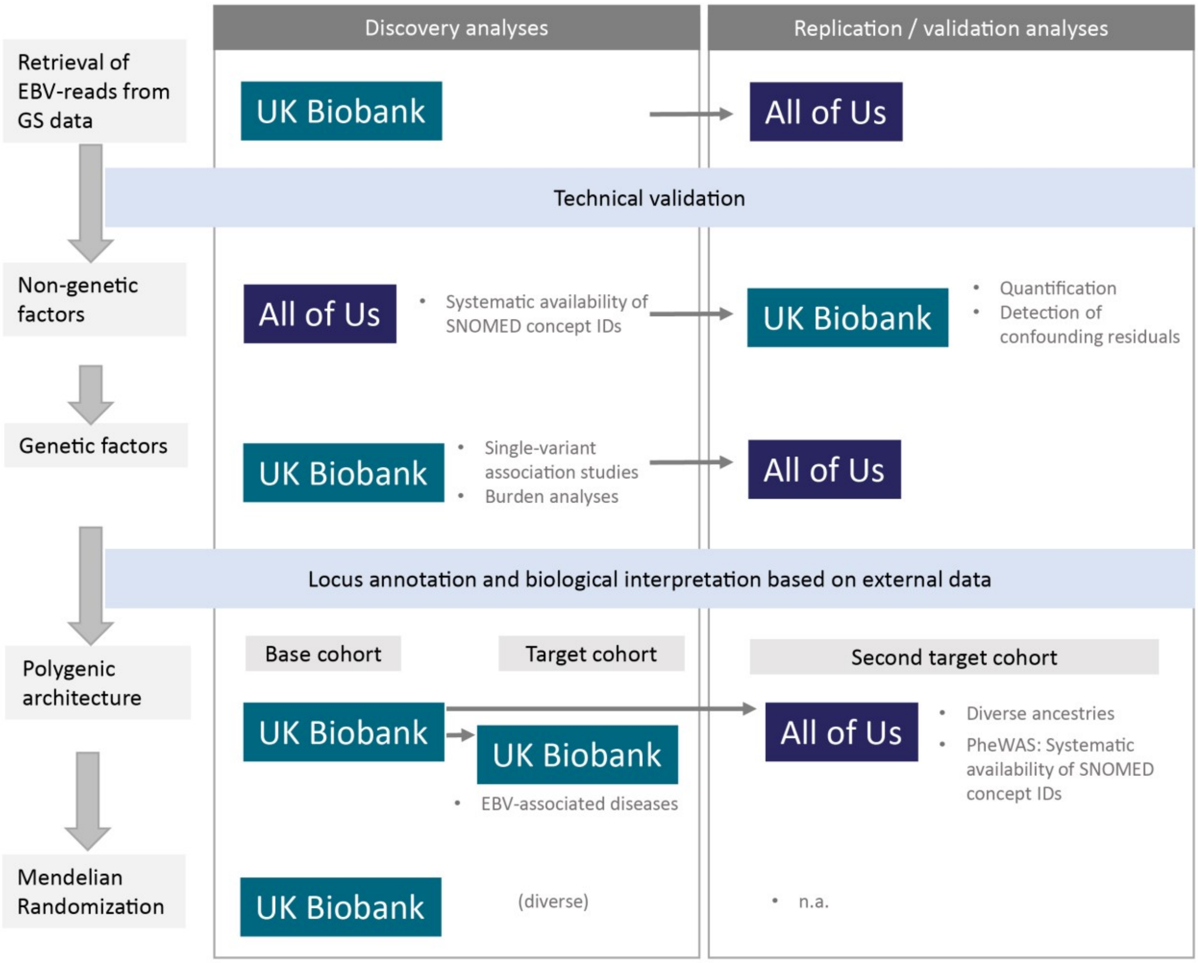

Supplementary Note 2: Extraction of EBV-reads in UK Biobank data

Analyses of individual-level data from the UK Biobank (UKB) were conducted within the UKB research analysis platform (RAP) using the frameworks R (v4.4.0), python (v3.9.16) and snakemake (v7.32.4). Plots were generated using ggplot (v3.5.1 within tidyverse 2.0.0) unless otherwise indicated.

DNA of UKB participants had been previously extracted from the buffy coat of whole blood. For genome sequencing, PCR free libraries were generated using the NEBNext Ultra™ II PCR-free kit (NEB) and sequencing was performed with Illumina´s NovaSeq6000 system on S4 flow cells (150 or 151 bp paired-end reads). Read alignment had been performed using Illumina DRAGEN (v3.7.8).

Identification of high-quality EBV-reads. Code snippets to extract and filter viral reads from CRAM files is provided below. Note that the file path to the CRAM-file (CRAM_FILE_PATH) needs to be set. The program version of samtools was 1.20. Filter settings determining high-quality reads were set by visual inspection of the aggregated alignment file of EBV-reads using the Integrative Genomics Viewer (IGV; v2.12.3), based on reads that showed evidence for unspecific mapping. We used the following code to extract EBV-reads (based on reference genome available in GRCh38):

```
samtools view -e "rnext==rname" -h "$CRAM_FILE_PATH" chrEBV > 
raw_reads.sam

samtools view raw_reads.sam -e 'sclen<=20 && rlen>=120' -F 1024 -O 
BAM -o filtered_reads.bam

samtools view filtered_reads.bam | cut -f1,4 > 
reads_mapping_to_EBV.tsv
```

Quality control. To exclude technical biases across library plates, we first calculated the proportion of individuals with EBV-reads per plate (field 32056). Upon inspection of the distribution (**Extended Data Figure 1**), individuals on library plates within 2 standard deviations of the mean (i.e., up to 28.8% EBVread positive samples per library plate) were considered as “regular plates”. We observed 51 library plates with higher fractions and split them into an „intermediate group“ (28.8% to 80%) and an „outlier group“ (>80%). We then called EBV variants using a multi-sample bam file of the aggregated EBV-reads across all groups (commands: mpileup (parameters: -a QS -r chrEBV -d 9999), call (parameters: -G - -mv) within bcftools (version 1.20)). From that file, we extracted all common variants with relatively high quality, i.e., only sites with an aggregated coverage above 300 and an allele frequency above 5% (n=2,240). Samples were then reattributed to the three groups (see above), and those variants that were not well represented in any of the three groups were removed from all three groups (defined as variants with an allele number (AN) below two standard deviations of the mean), resulting in n=1,687 variants. The same procedure was used to compare EBV-reads of individuals with one read and individuals with more than one read from the *UKB-QC*-cohort, which resulted in n=1,768 variants to be used for comparisons.

Analysis of outlier library plates: Visualisation of reads in IGV and the comparative analysis of allele frequency data indicated substantial variation within the EBV- genome in aggregated EBV reads from regular plates (**Extended Data Fig. 1; Supplementary Figure 1**). In contrast, the reads of the outlier group almost perfectly matched the EBV reference. In particular, we also observed a drop in coverage in a region between ~140,000 and ~150,000 bp (**Supplementary Figure 1**), indicating presence of a deletion within the EBV-genome from which the reads in the outlier group originated. These features point to a contamination with EBV from the B95-8 cell line, which was used to construct the EBV reference genome NC_007605.1. In NC_007605.1, only the region between ~140,000 and ~150,000 bp was

derived from another EBV strain, as this region is indeed deleted in EBV from B95-8. The presence of EBV from B95-8 might e.g. stem from Lymphoblastoid Cell Lines (LCLs)[98], as the B95-8 EBV strain is commonly used for generating LCLs. The intermediate plates have a mixed pattern, indicating that this group might contain both contaminated and non-contaminated plates. Based on a conservative approach, we removed all samples processed on the 51 plates belonging to the intermediate and outlier groups.

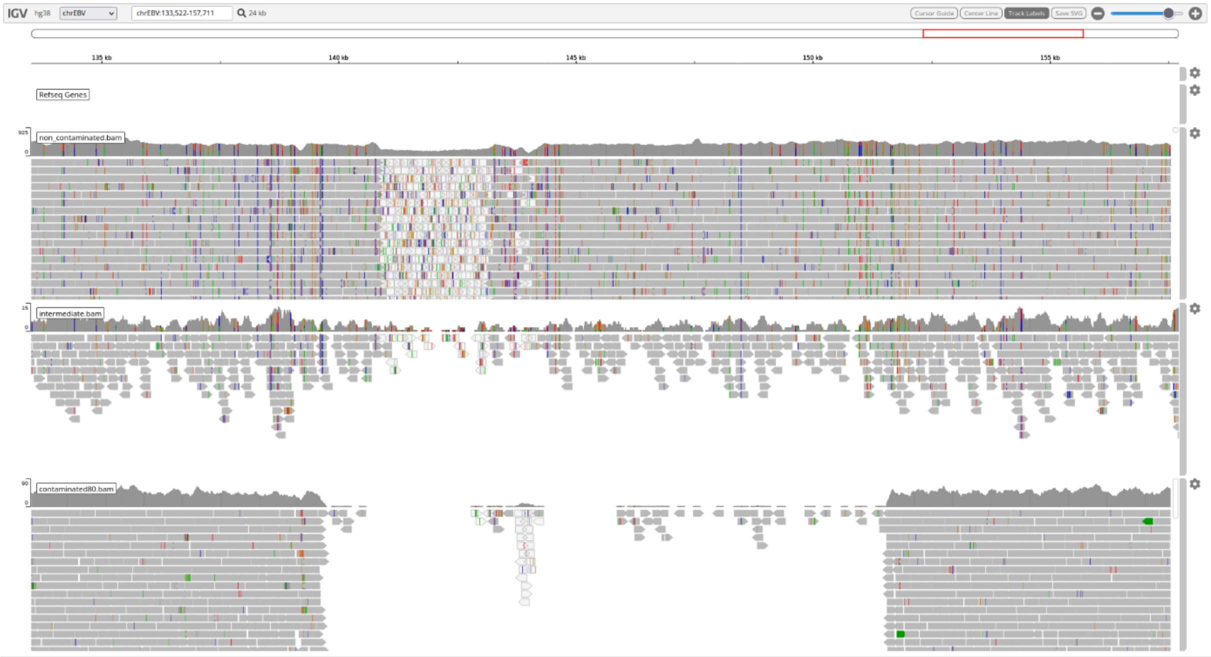

Supplementary Fig. 1: Aggregated EBV-reads of regular plates, intermediate plates and outlier plates. For each of the three groups, accumulated coverage and representative individual reads are shown in an IGV screenshot. The coverage/read data visualized at the top is taken from regular plates, the middle from intermediate plates, and the bottom from outlier plates, within the base-pair positions 133,522-157,711 of the EBV genome (NC_007605.1). Note the deleted region (~140,000 and ~150,000 bp) within the reads stemming from outlier plates.

Analysis of EBV-reads from EBV-read count=1 individuals: Specifically comparing individuals with EBVread count=1 and those with counts of 2 and more revealed (i) an identical distribution of reads (**Fig. 1c**), (ii) high correlations of allele frequencies and per-gene coverage (**Extended Data Fig. 2**), and (iii) considerable allelic variability distinct from the contaminated samples (see above, **Extended Data Fig. 2**). Together, this suggests that individuals in whom EBVread-counts are detected are likely to reflect true positives, even if identified with EBVread count of 1.

Supplementary Note 3: Additional information for AoU analyses

Definition of smoking- and HIV-status. Current smokers were defined as individuals that gave the answer "Some Days" or "Every Day" to the question "Do you now smoke cigarettes every day, some days, or not at all?" of the AoU Lifestyle questionnaire or as the presence of "Nicotine dependence" within the conditions. Individuals were annotated as having HIV if any of the following was true: (i) Answered "Yes" to the question "Are you still seeing a doctor or health care provider for HIV/AIDS?", (ii) gave a plausible answer to the question "About how old were you when you were first told you had HIV/AIDS?", or (iii) was annotated with any of the following conditions: Human immunodeficiency virus infection, Human immunodeficiency virus carrier, HIV-positive, Acute HIV infection, AIDS or AIDS-associated disorder.

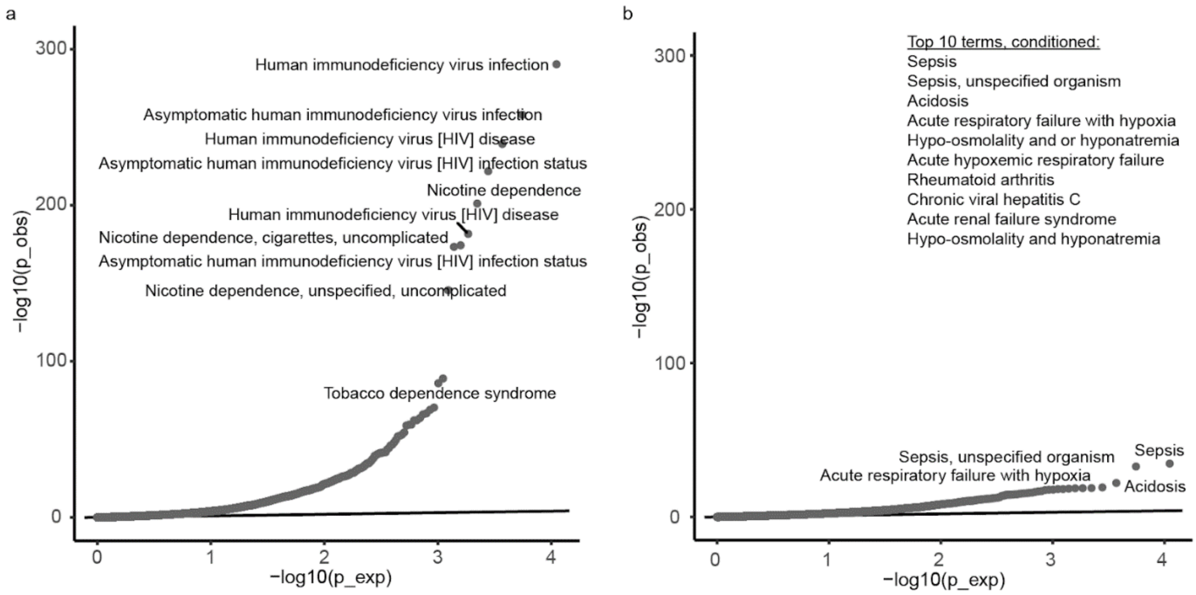

Supplementary Figure 2: QQ-plots of the association analysis between SNOMED concept IDs and EBVread+ status within All of Us. In the *AoU-QC*-cohort, associations of EBV-reads with 11,111 SNOMED concept IDs were tested. **a)** The negative decadic logarithms of P-values observed for the unconditioned analysis (y-axis) are plotted against the expected distribution (x-axis). In **b)** the analysis was repeated after conditioning on smoking- and HIV-status. For top10 and top4 SNOMED concept IDs, respectively, annotations are provided. All values are provided in **Supplementary Table S2**.

Variant sets in AoU: GS-based variant call sets were used to identify and analyse lead variants from the UKB GWAS. This data set was accessed as plink2 binary files, multiallelic sites were split into biallelic records using bcftools norm (v1.12). Non-imputed genotype data contained 1,739,269 variant sites and were accessed as plink 1 files, which were used for PCA and GRS analyses.

Principal component analysis within subcohorts of one genetic ancestry: PCA was calculated 20 PCs for each ancestry in analogy to the UKB procedure (**Supplementary Note 5**) based on genotyping data.

Supplementary Note 4: Analysis of covariates in UK Biobank

Preprocessing of covariates. Covariates of the *EUR-cohort* were preprocessed to ensure suitability for association testing (**Supplementary Table S4**). First, individuals with missing values in blood count traits, in fasting time prior to blood sampling, smoking status and time of blood sampling for the initial visit of the assessment center (instance 0) were removed from further analysis (n_{rm}=19,510). Note that we used lymphocyte percentage instead of the strongly correlated measure neutrophil percentage, in contrast to ref[2]. The number of cigarettes smoked per day (UKB data field 3456) or number of cigarettes previously smoked per day (current cigar/pipe smokers; data field 6183) was aggregated as “CigDayCurrent”. Missing values in current smokers were imputed with the mean of observed values ($n_{imputed}$=10,726). The number of alcoholic drinks per week (DrnkWk) was calculated from weekly alcoholic drinks or, if not available, from monthly drinks or alcohol intake frequency. If all values were missing, mean imputation was performed ($n_{imputed}$=233). Additionally, to ensure plausibility of covariates and in analogy to ref[2], outliers on four variables were winsorized at specific upper bounds: Fasting time value: 18; Pack years of smoking: 100; DrnkWk: 140; CigDayCurrent: 60. We excluded individuals with extremely high EBV-read counts (i.e. those above the 99th percentile within positives), as these individuals potentially have differing etiologies (**Supplementary Figure 3**):

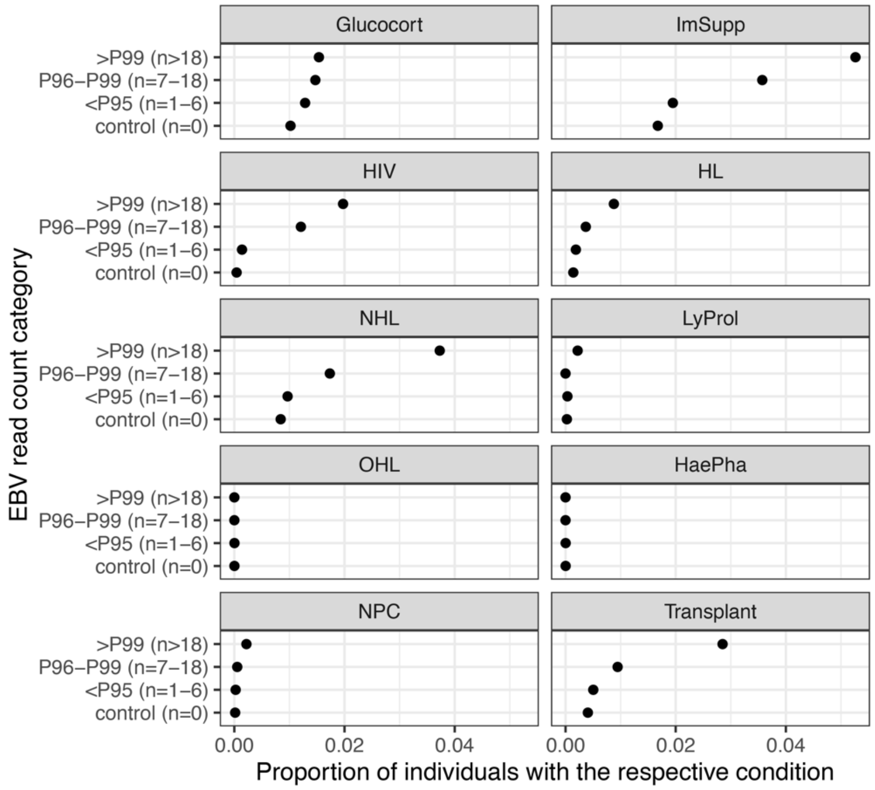

Supplementary Fig. 3: Distribution of covariates among individuals classified into different percentiles of quantitative EBV-reads. Individuals were grouped according to their EBV-read count (n) into a control group (n=0 reads, 264,616 individuals) or percentiles (P) within EBVread+ individuals (<P95: n=1-6 reads, 46,867 individuals; P96-P99: n=7-18 reads, 1,904 individuals; >P99, n>18,456 individuals). For each of 10 candidate conditions, data were retrieved from UKB data fields as described in **Supplementary Table S23**. This analysis shows that for the top-1% of EBV-read counts, the likelihood of some underlying malignancy increases. Therefore, individuals with 19 reads or higher were excluded from further systematic analyses. Point estimates based on n=313,843 unrelated individuals (*No-outlier-cohort* with the addition of individuals with read counts greater than 18). Abbreviations are as follows: Glucocort: Glucocorticoids; ImSupp: Immunosuppressive drugs; HIV: HIV infection; HL: Hodgkin Lymphoma, NHL: Non-Hodgkin Lymphoma; LyProl: Lymphoproliferative disorder; OHL: Oral hairy leukoplakia; HaePha: Hemophagocytic lymphohistiocytosis; NPC: Nasopharyngeal Carcinoma.

Next, individuals were excluded based on additional outlier measures: (i) individuals with very early or very late date of recruitment (outside of the range April 2007 to July 2010, n_{rm}=849), (ii) individuals with outliers in blood count traits, i.e., any blood measurement with a Z-score > 4, based on ref[71] (n_{rm}=11,522). These steps lead to the *No-outlier*-cohort. Covariates that could not be reasonably approximated by a linear relationship with respect to the presence of EBV or HHV-7 reads were modeled with natural splines (R library splines, v4.4). The natural splines were fit on the unrelated subset of individuals, for the following covariates and using the degrees of freedom (df) given in parentheses: date of attending assessment center (df=7); month of attending assessment center (df=3); time blood sample collected (df=5). With the fitted natural spline models, we calculated the chance of observing EBV or HHV-7 reads for each individual and each of the aforementioned covariates, respectively.

Selection and effects of covariates. To define the most relevant set of the 28 covariates, we used forward and backward selection with Bayesian information criterion (BIC) as a quality parameter (stepAIC function of the 'MASS' library, v7.3-61 within R) on the *No-immune-supp*.-cohort. The phenotypes "EBVread+" and "HHV-7read+" were used as outcomes, respectively, and covariates were retained for the use in our genome-wide association studies (GWAS) if they were selected by one of the two analyses. The 18 covariates finally selected for GWAS are given in **Supplementary Table S4.** Further, the effects of changes in individual covariates were determined using marginal standardization[99]. To obtain covariate distributions, marginal standardization was applied to each of 1000 resamples (bootstrapping). Only non-related individuals of the *No-outlier*-cohort (n= 313,843) or *No-immune-supp*-cohort (n= 305,544) were used.

Impact of covariate selection on GWAS results. Data from a recent biobank-based study, on mtDNA load in blood[71], suggested that differences in covariates between biobanks might introduce biases, and wrong conclusions. In particular it was described that not-accounting for blood count traits can lead to confounded results when analyzing blood-based measures, such as mtDNA copy number. To investigate the effect of accounting for different sets of confounders in a controlled setting, we re-ran the UKB analyses, excluding either blood count traits or all covariates beyond age, sex and PCs (**Supplementary Table S3**), and performed correlation analyses for the genome-wide significant variants from the EBVread+ GWAS. Interestingly, the effect of blood count traits as well as additional covariates beyond age, sex and PCs as covariates was smaller than assumed, at least for the genome-wide significant loci/variants (**Extended Data Figure 7**). However, we cannot rule out any residual confounding beyond the genome-wide significant findings. In order to follow a conservative approach for reporting our result and in analogy to Gupta et al[71], we therefore kept the genetic analysis in AoU to a replication-only strategy. Still, a meta-analysis would likely identify more genetic contributors to EBV viral load control, and including more diverse ancestries could also be used for more specific finemapping.

Supplementary Note 5: Analysis of JCTF data (paired GS/bulk RNA seq)

The paired GS/bulk RNA-seq data used for correlation of EBV-transcript count with EBV-read counts were obtained from samples collected at the time of or following SARS-CoV-2 infection[26]. To analyze whether the results are influenced by different degrees of COVID-19 severity, we re-analyzed the data with respect to their individual COVID-19 disease course (**Supplementary Figure 4**). Overall, phenotype information was available for 1,007 (out of 1,010) individuals who were asymptomatic (n=72), had mild symptoms (n=259), had severe COVID-19 (requiring oxygen support, n=378) or very severe COVID-19 (intensive care unit and ventilated; n=298). First, EBV-read counts and EBV-transcript counts were plotted against each other and coloured according to COVID-19 severity. We observed that individuals with high EBV-read counts (>10) had a variety of COVID-19 degrees of severity, including mild, severe and very severe disease course (**a**). Among 331 individuals who were either asymptomatic or mildly affected with COVID-19, 103 were EBVread+, which corresponds to 31.1% (as opposed to 39.2% in the overall JCTF cohort). We also observed individuals with EBV-read counts > 10 in this asymptomatic/mild subcohort **(b).** The fraction of individuals in whom EBV-transcripts were detected, was also similar to the overall cohort (**(c),** cf. Main Figure 1g).

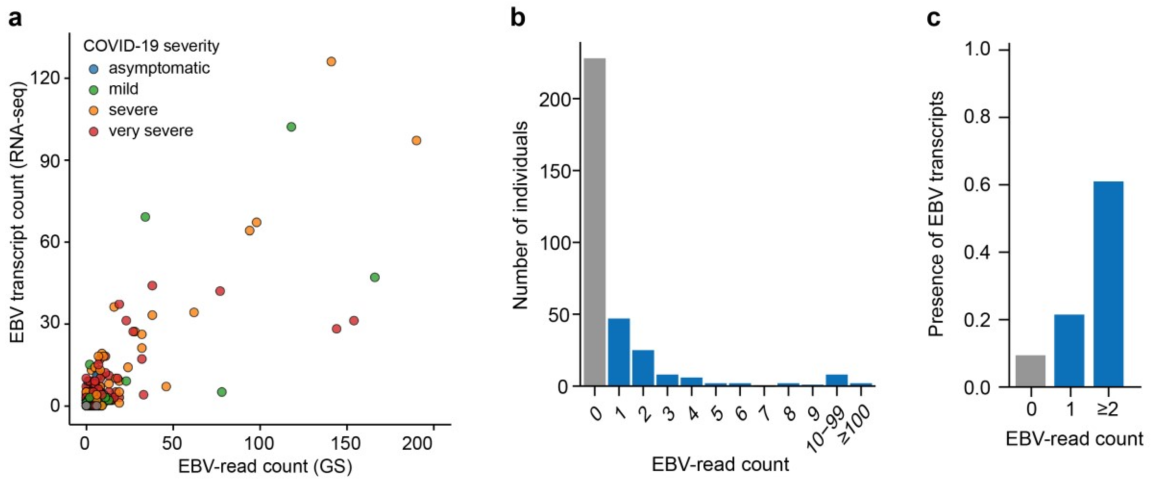

Supplementary Figure 4: Effect of COVID-19 severity on EBV-read and EBV-transcript counts. (a) EBV-read counts are plotted against EBV-transcript counts, for 1,007 individuals of the JCTF (colors represent different COVID-19 severity). Individuals of different severities are present among those with higher EBV-read counts. **(b)** The distribution of GS-based EBV-reads in the mild/asymptomatic individuals (n=331) is similar to the overall distribution in the entire cohort, though the fraction of EBVread+ individuals (in blue) is lower. **(c)** For asymptomatic/mild individuals within different EBV-read count groups (0, 1, or 2 and higher), the fraction of individuals with EBV-transcript counts is depicted. Again, the distribution is similar to the overall cohort.

We finally calculated the effect of age, sex and COVID-19 severity in the entire cohort of 1,010 individuals, using multivariate logistic regression. For both GS-based EBV-read counts and RNA-seq based EBV-transcript counts, respectively, we found that age is significantly associated, while sex and COVID-19 severity is not (**Supplementary Table S5**).

Supplementary Note 6: Extraction of HHV7-reads in UK Biobank data

For HHV7 no reference sequence is contained within GRCh38. Therefore, we extracted unmapped reads from the GS CRAM files and aligned them to an HHV7 fasta file based on NCBI data (NC_001716.2; given as fasta file VIRUS_REFERENCE_SEQS.fasta), using bwa-mem2 (v2.2.1). Read filtering was performed using samtools version 1.20. Visual inspection of aggregated HHV7 reads in IGV after applying the same criteria as for EBV yielded residual unspecific mapping. Therefore, the filters for sequencing reads were set to stricter criteria as compared to EBV and we only kept reads that met the following criteria: forward and reverse sequences mapping to HHV7, non-zero mapping quality, 10 or less soft-clip bases and 130 or more reference bases, not marked as duplicate. Finally, only read pairs were counted whose forward and reverse read passed these filters.
This resulted in the following code for HHV7:

```
bwa-mem2 index VIRUS_REFERENCE_SEQS.fasta

samtools view -f 12 -u "$CRAM_FILE_PATH" "*" | samtools fastq -1
FASTQ_FILE_1.fq.gz -2 FASTQ_FILE_2.fq.gz -0 /dev/null -s /dev/null -
n

bwa-mem2 mem VIRUS_REFERENCE_SEQS.fasta FASTQ_FILE_1.fq.gz
FASTQ_FILE_2.fq.gz | samtools view -F 12 -h > VIRUS_READS.sam

samtools view VIRUS_READS.sam -h -q 1 -e 'sclen<=10 && rlen>=130 &&
rnext==rname' -F 1024 | samtools view -f1 -O BAM -o
VIRUS_READS_FILTERED.bam

samtools view VIRUS_READS_FILTERED.bam | cut -f1,3,4 >
reads_mapping_to_viruses.tsv
```

Similar to EBV, we performed quality controls and compared the HHV7-results from GS data to serology data. Results are depicted in **Supp. Figure 5**.

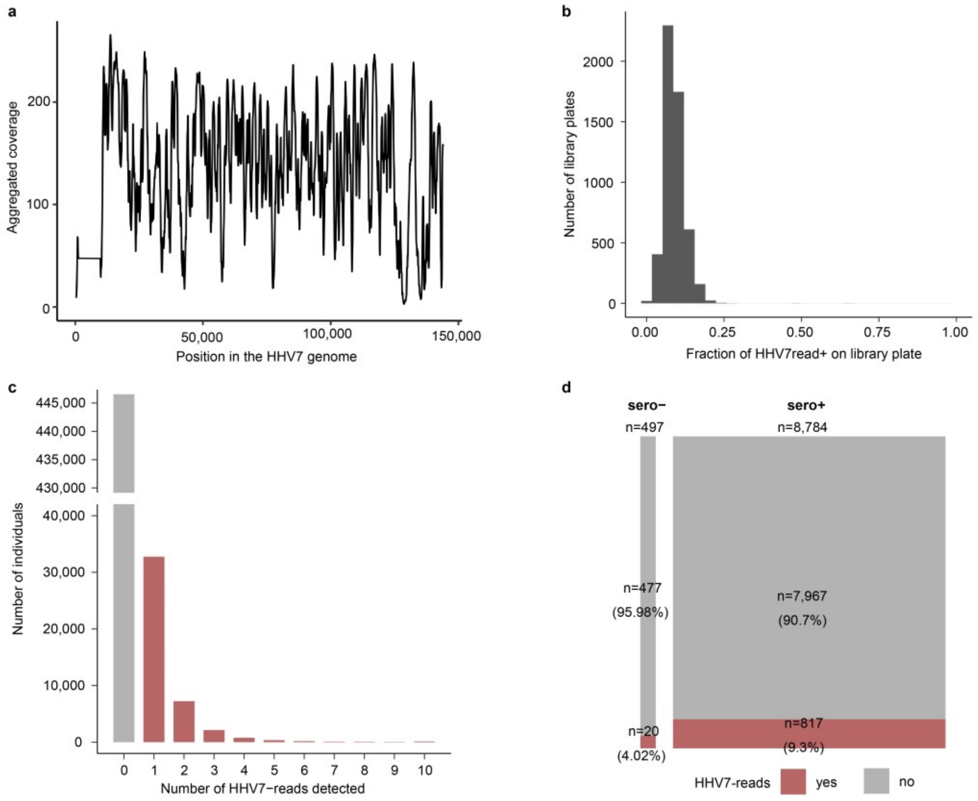

Supplementary Fig. 5: HHV7-read extraction within UKB. a) Cumulative read coverage across the HHV7-genome based on the *UKB-QC*-cohort (n=486,315 individuals; line smoothed, rolling windows of 500 bp). **b)** Quality control of library plates, illustrating the distribution of HHV-7-read+ individuals per library 96-well plate. Differently from EBV, no clear indications of contaminations were observed. **c)** Number of HHV7-reads detected per individual, with individuals showing at least one EBV-read highlighted in red (HHV7read+). For illustration purposes, the y-axis has been interrupted between 42,000 and 430,000 individuals. **d)** Correlation of HHV-7read+ status with HHV7 serology data (HHV7sero-negative (sero-) and positive (sero+)), determined in the subset of the *UKB-QC*-cohort with HHV7 serology data (data field 23058).

Supplementary Note 7: Monogenic susceptibility for EBV infection

Among the nine IEI genes with test-wide significance in the MAGMA gene analysis, we found four to be associated with IEIs that have marked and well-documented susceptibility to EBV infection or chronic/ clinically more severe manifestations of EBV infection, including *IKZF3/Aiolos* deficiency[100], *NFKB1* deficiency[101], *CTLA4* deficiency[102-104] and, most profoundly, CD70 deficiency[59,60,105]. These four IEIs are also associated with increased susceptibility for lymphoma, often EBV-driven. This is in line with the clinically accepted notion that inability to clear an active EBV infection is associated with significant risk of lymphoma development. Regarding the risk for other infections, this is well documented for two genes, while it has not yet been shown for the two others (though it is plausible based on the pathomechanisms of the associated deficiencies).

For three additional genes, it is at least plausible that they would also go along with clinically more profound EBV infection: *DCLRE1B*, in which loss-of-function mutations cause a dyskeratosis congenita phenotype with bone marrow failure including B- and NK-cell lymphopenia as well as T-cell dysfunction[106], *IKZF1/Ikaros* (described as dominant-negative or gain-of-function mutations, respectively[107-109], and *IRF1* (whose deficiency that causes NK cell lymphopenia and decreased type 1 dendritic cells[110]. Similarly, it seems plausible that these genes are involved in increased risk for both other herpes infections and associated cancers, though this has not yet been documented.

The IEIs caused by the two genes *CCR2*[111] and *ICOS*[112] have not yet been reported with any relation to EBV.

Supplementary Note 8: Additional information on Two-sample MR

Results for rheumatoid arthritis (RA) and type 1 diabetes (T1D) showed significant effects across all estimators, including the robust estimators MR-RAPS (OR = 1.67, 95% CI = [1.47, 1.91] and OR = 1.90, 95% CI = [1.72, 2.11], for RA and T1D, respectively) and MR-PRESSO (OR = 1.34, 95% CI = [1.04, 1.72] and OR = 1.62, 95% CI = [1.31, 2.00], respectively). Notably, these pleiotropy robust estimators showed significant effects despite the presence of significant horizontal pleiotropy for the T1D analysis (MR-Egger intercept = –0.04, *s.e.* = 0.001, *P* = 0.001). However, all these effects also attenuated towards null when restricting to non-MHC SNPs (e.g., RA: OR_{wMed} = 1.06, 95% CI = [0.84, 1.34]; T1D: OR_{wMed} = 0.96, 95% CI = [0.67, 1.40]). The negative control trait (hair colour: red) did not show any significant effects (e.g., OR_{wMed} = 1.02, 95% CI = [0.97, 1.08]).

.

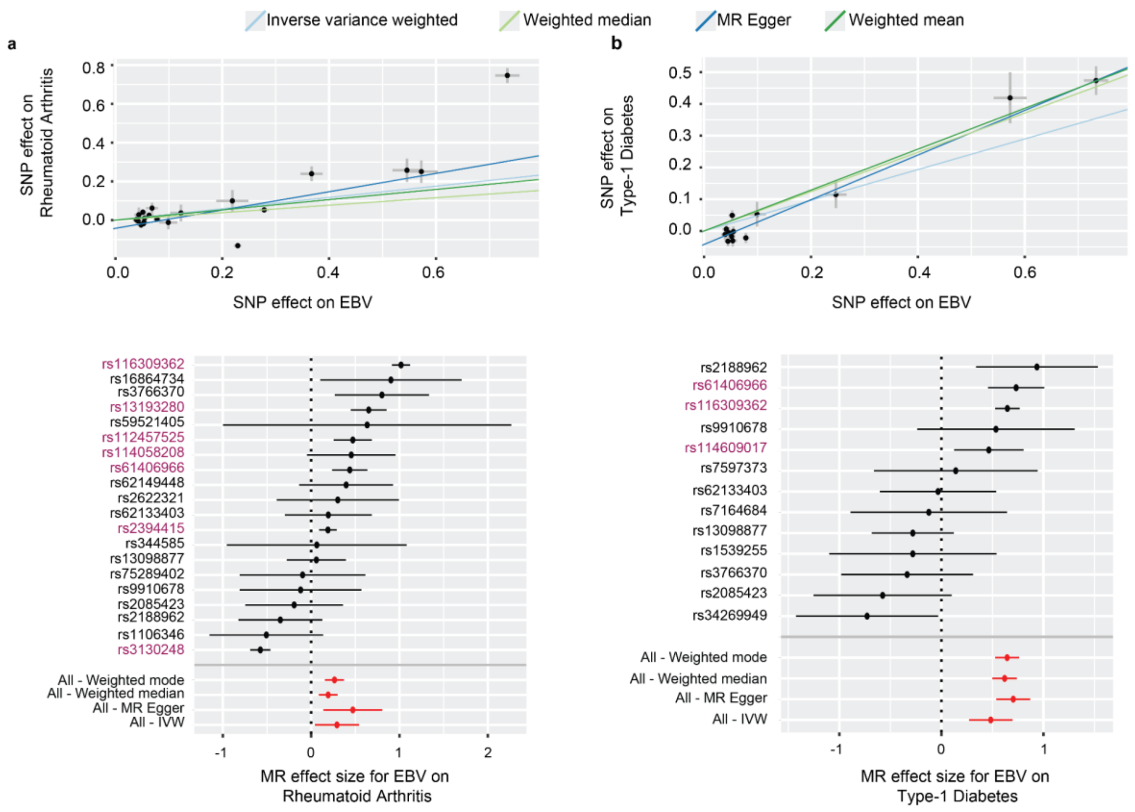

Supplementary Figure 6: 2SMR results for rheumatoid arthritis and type 1 diabetes. The figure shows results of MR testing of EBV-read+ (exposure) on Rheumatoid arthritis **(a)** and Type 1 diabetes (T1D; **b)**. Outcome summary statistics were obtained from GWAS catalogue for RA (GCST90132223, EUR, 22,350 cases, 74,823 controls) and T1D (GCST90013446, EUR, 13,458 cases, 20,143 controls). Upper panel: scatter plots using the four standard estimators. 'SNP effect' refers to beta-values as obtained from summary statistics (point), and error bars refer to standard error. Lower panel: forest plots (black line: effect of individual variants, red line: overall effect estimates. Variants at MHC are highlighted in purple).

Supplementary Note 9: Generation of genetic variant sets in UK Biobank data

Variant set for quality control (QC)/PCA. We generated a high quality set of common variants for 403,192 individuals using plink v1.90b7.4[113], by selecting genotyped variants (UKB field 22418) matching the following conditions: Variant minor allele frequency (MAF) above 1%, variant level missingness below 5%, Hardy-Weinberg p-value (midp-correction) above 1E-25, location on chromosome 1-22 and outside of long-range linkage disequilibrium (LD) regions (https://genome.sph.umich.edu/wiki/Regions_of_high_linkage_disequilibrium_(LD)). Finally, variants were pruned based on LD (plink parameter --indep-pairwise 1000 50 0.05), which resulted in a set of 80,217 variants. Within this variant set, individuals with a variant-missingness > 5% were removed (n_{rm}=172 individuals). Related individuals (kinship-coefficients ≥ 0.0442) were identified according to the file "Genotype Results/Genotype calls/ukb_rel.dat" as provided by the UKB.

To obtain an unrelated subcohort, related individuals were iteratively filtered out, starting with individuals with the highest numbers of relations ("non-rel." subsets, see **Fig. 1a**). PCA was then conducted using FlashPCA (v2.0)[114], on the unrelated subset of the cohort, while related individuals were subsequently projected onto the same principal component space. For regenie step 1, a slightly different variant set was obtained in analogy to the filter steps described for PCA, except that the MAF-threshold was set to 0.1% and the settings for pruning were relaxed (plink parameters: --indep-pairwise 1000 50 0.2). This resulted in 279,126 variants for 403,029 individuals. In total, 403,014 individuals had both principal components calculated and step1 variants available (*EUR*-cohort).

Variant set for rare-variant tests. UKB exome sequencing and secondary analysis of sequencing data is described elsewhere[74]. In short, IDT's xGen probe library was used for library preparation and sequencing was conducted as 75-base-pair paired end sequencing on Illumina NovaSeq 6000 platforms using S2 or S4 flow cells. The main steps of the secondary analysis comprised alignment to GRCh38 using bwa mem (v0.7.17), variant calling using deep variant (v0.10.0) and joint calling with GLnexus (v1.2.6). From this call set we used variant sites where 90% of individuals had a coverage of at least 10x. For variant annotation, this was created using SnpEff with Ensembl v85 as the underlying database. Missense variants were classified based on the number of missense prediction scores indicating a deleterious effect: 5/5 – 'likely deleterious', 1–4/5 – 'possibly deleterious', and 0/5 – 'likely benign'. The scores used were SIFT, PolyPhen2 HDIV, PolyPhen2 HVAR, LRT, and MutationTaster. Pre-computed variant annotations including assignments to genes were retrieved from the UKB RAP (file: ukb23158_500k_OQFE.annotations.txt.gz), based on ref[74].

Supplementary Note 10: Analysis of MHC region with and without SPA

In the final EBVread+ summary statistics, we observed that P-values for variants at the MHC region reached a plateau at -log10(P) of 306.653, likely reflecting statistical limitations of the SPA for extremely strong associations. We repeated the SNP-based association analysis for the MHC region (25Mb - 36Mb, chrom. 6) without SPA, with the aim to get unbounded P-value estimates. Indeed, rerunning the GWAS without SPA for the MHC region revealed P-value estimates for all variants above the plateau, with all other P-values remaining strongly correlated and effect sizes remaining nearly identical for all variants with or without SPA (**Supplementary Fig. 7**).

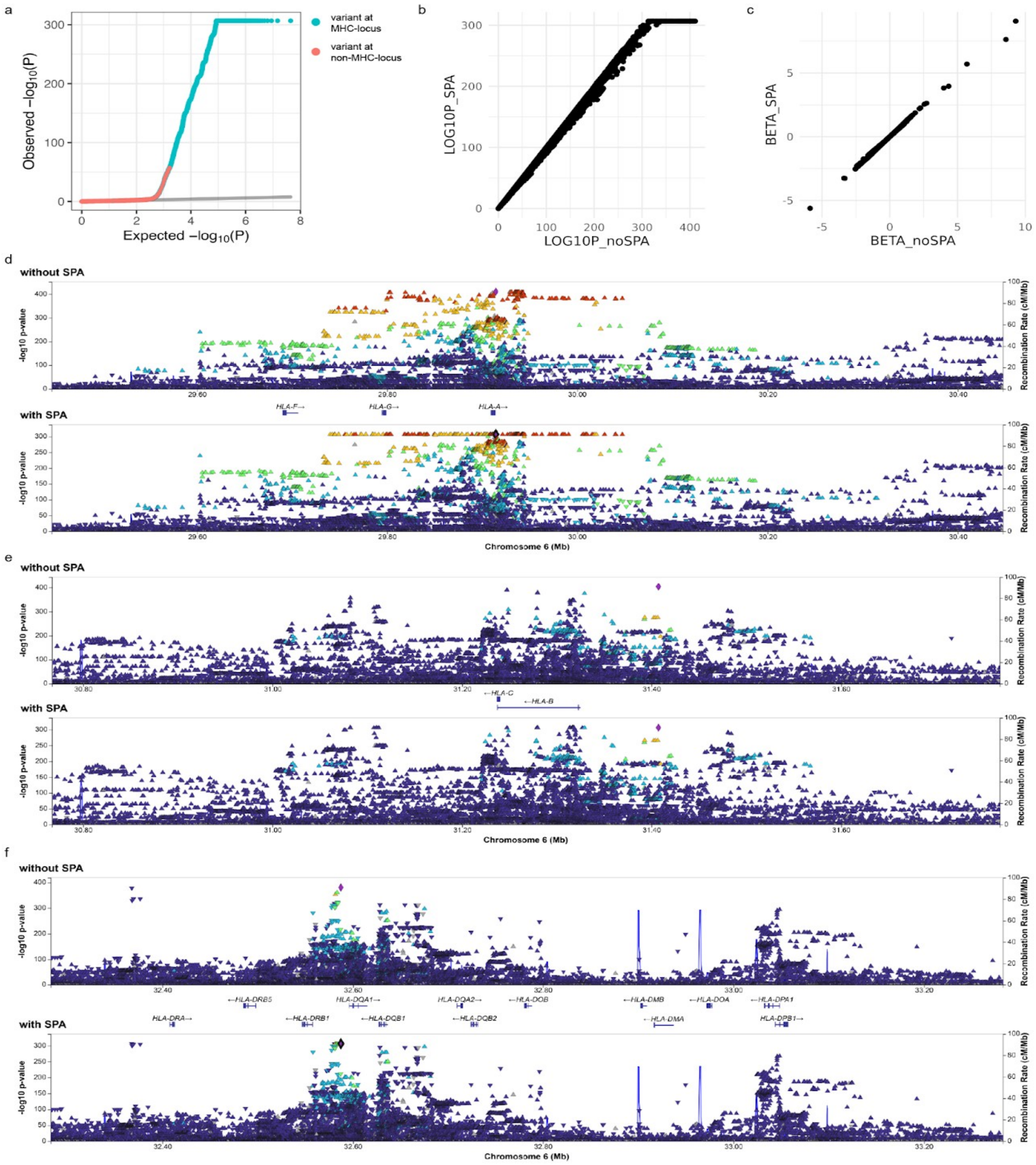

Supplementary Figure 7: Effect of saddle-point approximation (SPA) on P-values in the MHC region. (a) QQ-plot when conducting the main GWAS on EBVread+ using Saddle-Point-Approximation (SPA; UKB-no-immune-supp-cohort: 360,283 individuals, including 56,180 cases and 304,103 controls). Test statistics were slightly inflated (λ=1.048, LD Score regression intercept: 1.0265), which was partially attributed to the highly significant associations identified at the major histocompatibility complex (MHC) locus (without MHC region (chr6: 25-36Mb): λ=1.039). **(b)** Scatter plot of negative decadic logarithms of p-values generated by regenie, with and without SPA for the MHC region (25-36 Mb). We noted that the correction of P-values by SPA that we used to counteract case-control imbalances caused a ceiling of p-values at -log10(p) of 306.653 for the most significant variants. We therefore replaced these -log10(p) values by the corresponding uncorrected values without SPA. **c)** Betas of MHC-variants with p<0.05 were highly correlated (r>0.9997) between the analyses with or without SPA

(Pearson's correlation; two-sided $P<2.2 \times 10^{-16}$). Panels **(d)** to **(f)** show the regional association plots for variants at the MHC region with (bottom panel) and without SPA (top panel), respectively, divided into regions approx. 1 Mb. In between the panels, HLA-genes are shown, respectively. Plots generated with Locus Zoom, using the European population as reference.

Supplementary Note 11: Epistatic analysis

Interaction was analyzed within the *UKB-no-immune-supp*-cohort (minus individuals who withdrew consent during study (n=115) or had missing genotype dosages (n=594), leaving n=304,523) between the 54 conditionally independent HLA-alleles and lead variants at the three top non-MHC loci. This selection was made to reduce the multiple testing burden, and was further informed by the fact that only *ERAP2*, among all candidate genes, has a prior role in antigen processing. The following models were used:

Additive model: $logit(P(EBVread+)) \sim \beta_0 + \beta_1\ SNP + \beta_2\ HLA + \beta_{cov}\ cov$

Interaction model: $logit(P(EBVread+)) \sim \beta_0 + \beta_1\ SNP + \beta_2\ HLA + \beta_3\ SNP*HLA + \beta_{cov}\ cov$

In these models, EBVread+ is the binary presence of EBV reads, SNP is the continuous non-MHC SNP effect allele dosage, HLA is the continuous HLA effect allele dosage, β_0, β_1, β_2, β_3 are the scalar regression coefficients of the intercept, SNP effect, HLA effect, and SNP-HLA interaction effect, respectively, cov is the matrix of included covariates and β_{cov} is the vector of regression coefficients of the included covariates.

Supplementary Note 12: HLA imputation in AoU

HLA imputation: While information on HLA-alleles were readily available in UKB, this is not the case in AoU and required imputation of HLA genes using the HLA-TAPAS framework[115]. Therefore we created a reference panel for the AoU cohort using the MakeReference module of HLA-TAPAS, with genotype data of the 1000 genomes project phase 3 (1000Gv3; n=2,504 individuals) and accompanying HLA-alleles (provided by HLA-TAPAS). HLA-alleles were then imputed with the SNP2HLA module of HLA-TAPAS, using genotyping array data and batches of 2000 individuals.

QC of HLA imputation using HLA typing: 150 individuals of each of the six ancestries within All of Us were randomly sampled. Then the software kourami, version 0.9.6[116] was used to type HLA-alleles from CRAM files. This yielded HLA-alleles for the genes HLA-A, HLA-B, HLA-C, HLA-DQA1, HLA-DQB1, HLA-DRB1 across 888 individuals (12 individuals failed HLA typing). We required that HLA-typing successfully typed two alleles per gene per individual, otherwise, this gene/individual combination was removed. The resolution of HLA-alleles was reduced to two-field alleles. When typing yielded G-group alleles, these were mapped to two-field HLA-alleles using the mapping provided by the IPD-IMGT/HLA database (version 3620). Subsequently, imputed and typed HLA-alleles were compared on the level of two-field HLA-alleles (**Supplementary Fig. 8**). In particular, correlations between imputed and typed HLA-alleles were calculated using the r^2 metrics as previously described[117]. Additionally, Positive Predictive Values (PPVs) for imputed alleles were calculated using the following logic: For each HLA-allele predicted by imputation, it was checked if this allele was also found by HLA-typing. For each allele, we counted the number of alleles predicted by imputation ($n_{imputed,allele}$) and those that were also confirmed by typing ($n_{confirmed,allele}$). We then summed the number of imputed () and confirmed alleles for each gene ($n_{imputed,gene}$=allele genen$_{imputed,allele}$and $n_{confirmed,gene}$=allele genen$_{confirmed,allele}$). The gene-based PPV was calculated as the ratio $n_{confirmed,gene}$ / $n_{imputed,gene}$. A dosage between 0.5-1.5 was considered to represent one (heterozygous) allele, a dosage above 1.5 was considered to represent two (homozygous) alleles within the calculation of the PPV.

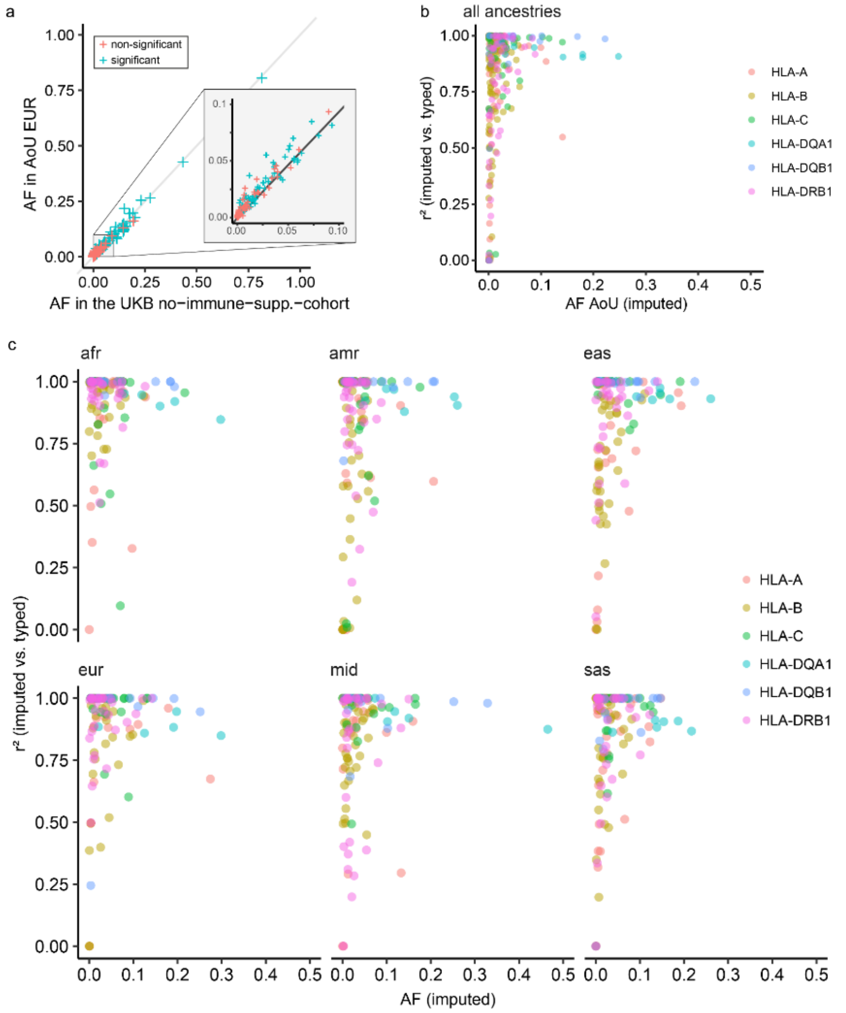

Supplementary Figure 8: Comparison of results from HLA allele imputation and typing in All of Us. (a) For each of the available HLA-alleles in UKB, the allele frequency (AF) in UKB (n=360,764 individuals) is plotted on the x-axis, against the corresponding AF in AoU EUR (n=184,948 individuals). Colors divide the data points by genome-wide significance (red: no; blue: yes), and the inlet increases visibility for the HLA alleles with less than 10% AF. **(b)**+**(c)**: Results of correlation analyses between imputed and typed HLA-alleles, for 888 individuals in AoU. Colors indicate different groups of HLA-genes. Plots include all 888 individuals in (b), and divided by ancestry in (c): afr: African (n=149 individuals); amr: Admixed American (n=148), eas: East Asian (n=149), eur: European (n=145), mid: Middle Eastern (n=147), sas: South Asian (n=150).

Supplementary Note 13: Details on the validation cohorts

i) Validation 1, qPCR - vARIANCE study. The vARIANCE study run at the University Hospital Bonn investigates genetic and non-genetic risk factors associated with ACE inhibitor-induced angioedema[25]. All 110 participants selected for the validation study had provided biological samples (EDTA blood: n=95; saliva: n=15), written informed consent, including explicit permission for their samples and data to be used as controls, and completed a questionnaire capturing baseline characteristics (**Supplementary Table S1**). The study protocol was approved by the Ethics Committee of the Medical Faculty of the University of Bonn (approval no. 101/16). Library preparation was performed using the NEBNext Ultra II FS DNA PCR-Free Library Prep Kit, with subsequent sequencing on NovaSeq6000. Sequence alignment against GRCh38 was performed using NVIDIA parabricks. EBV-read counts were extracted similarly to UKB. Subsequent qPCR was performed on 72 DNA samples from the same collection timepoint. These 72 samples contained all samples who were EBVread+ (excluding one for which no DNA was available; n=28), plus 44 randomly selected EBVread- samples. qPCR was performed using the GeneProofTM EBV PCR Kit (TaqPathTM Menu, Applied Biosystems) and a LightCycler® 2.0 / 480 (Roche), in a total volume of 20 µl, using 4.5 µl of DNA (100 ng/µl), calibrators or H_2O as negative control. 0.5 µl Internal Standard was added as PCR inhibition control. Each sample was measured in quadruplicates. As most samples with low EBV read counts (≤4) were around the limit of detection and below the range of calibrators provided in the kit (10 – 10,000 copies/µl, 1st WHO International Standard for Epstein–Barr Virus for Nucleic Acid Amplification Techniques, EBV NIBSC 09/260), we report the percentage of positive replicates (Cp<40) and average Cp values as a measure for EBV viral load.

ii) Validation 2: JCTF cohort. The JCTF cohort comprises individuals infected with SARS-CoV-2 at time of recruitment, who had different COVID-19 severity outcomes and spanned a wide range of ages. Details on recruitment strategies can be found in ref[75]. Among others, participants provided whole blood samples from which DNA was extracted for genetic analyses including GS. Briefly, GS libraries were constructed using the TruSeq DNA PCR-Free Library Preparation Kit (Illumina, CA, USA) or KAPA HyperPrep PCR-free Kit (KAPA Biosystems, MA, USA), and were sequenced on the NovaSeq 6000 (Illumina) system (2×150bp, average coverage: 27.6x). Sequenced GS reads were aligned against the GRCh38 human reference genome, including chrEBV, using bwa-mem (v0.7.15). Duplicated reads were removed using GATK MarkDuplicates (v4.1.0.0). EBV-reads were extracted from BAM files using the same computational protocol as for UKB/AoU. Based on the GS results, two subcohorts were designed to address the following validation aspects: a) to technically validate the GS-based EBV-read counts by qPCR, and b) to correlate GS-based EBV-reads with transcriptome data. Please note that there was only a limited overlap between samples used for a) and b).

a) qPCR sample. For 262 individuals, qPCR was performed to detect EBV genomic DNA using the QuantStudio 3 Real-Time PCR System (Applied Biosystems) and PowerUp SYBR Green Master Mix (Thermo Fisher Scientific, Cat. No. A25777), on the same aliquots than the GS data were generated. A synthetic DNA standard for *EBNA1* was prepared at six serial dilutions (1×10^6–1×10^1 copies for *EBNA1*; 2.5×10^6–2.5×10^1 copies for *BALF5*) as previously described[118,119]. Each 20 µL reaction contained 10 µL of 2× Master Mix, 1 µL each of forward and reverse primers (10 pmol/µL), 4 µL of nuclease-free water, and 4 µL of DNA template. Thermal cycling conditions were: UDG activation at 50 °C for 2 min, Dual-Lock DNA polymerase activation at 95 °C for 2 min, followed by 40 cycles of 95 °C for 15 s and 60 °C for 1 min. Melt-curve analysis (95 °C for 15 s, 60 °C for 1 min, 95 °C for 15 s) confirmed amplification specificity. Fluorescence was measured at each extension step. Assay validity was confirmed by amplification efficiency (90–110%), $R^2 > 0.99$, and a single melt peak. Standard curves were generated in parallel to quantify copy numbers for each gene.

b) RNA-seq analysis. For n=1,010 individuals, paired GS and transcriptome data (by RNA-seq) were available[26,120]; cohort characteristics in **Supplementary Table S2**). RNA-seq was performed at 2×100 bp on NovaSeq6000, and RNA-seq data were first aligned to the GRCh38 human reference genome, including chrEBV, using STAR (v.2.5.3a). Subsequently, EBV transcripts were quantified using RSEM (v.1.3.0), as previously described[120], with EBV gene transfer format (GTF; NC_007605.1). The sum of all detected EBV-transcript counts was analyzed as “EBV-transcript count”. SARS-CoV-2 infection and COVID-19 severity might both have an impact on EBVread+ status. However, at least for COVID-19 severity status, which was readily available, we did not detect a strong effect on EBVread+ (**Supplementary Note 5**).

Supplementary Note 14: Annotation of 27 non-MHC risk loci

For each locus, regional association plots (RAPs) were generated with Locus Zoom and are shown in the left column, based on the GWAS summary statistics from EBVread+ in UKB. In the right column, Forest plots provide information on the association statistics for the lead SNP of EBVread+ (unless otherwise indicated), and for HHV7 and memory B cells (see Methods).

Chr. 1p13.2_PTPN22

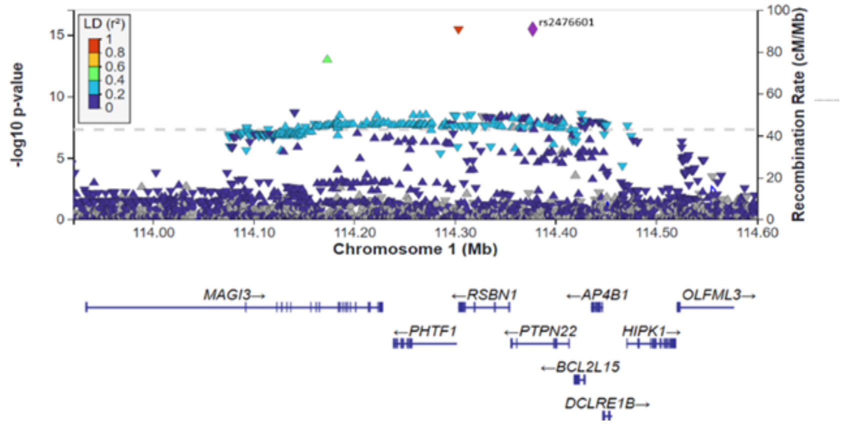

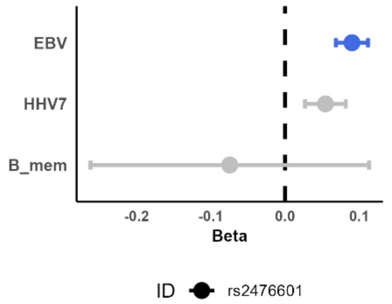

Chr. 1q23.3_SLAMF7

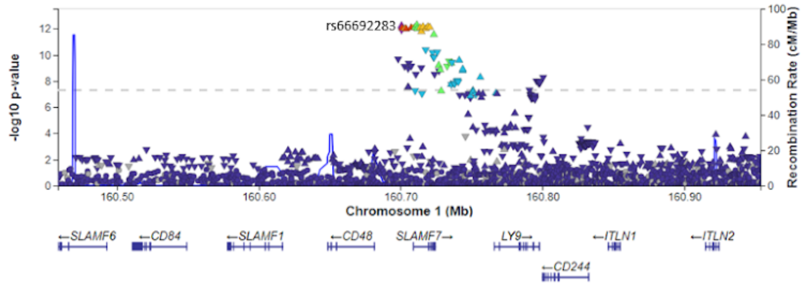

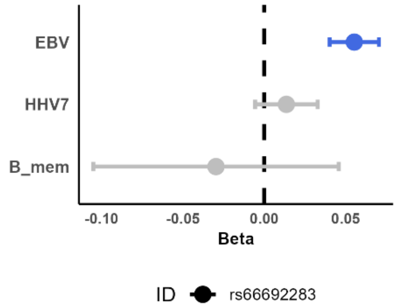

Chr. 1q25.1_PRDX6

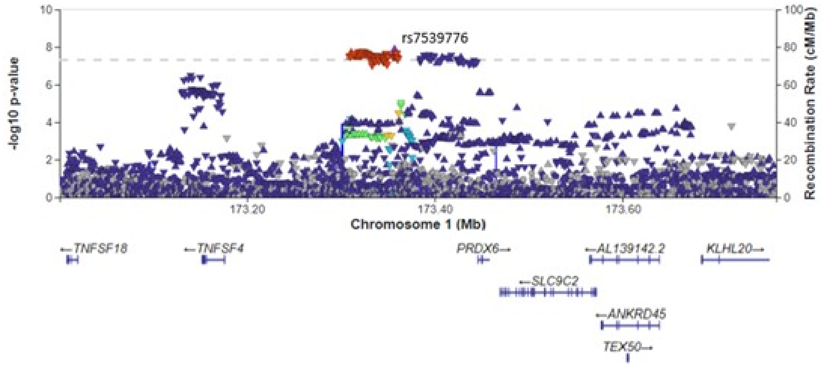

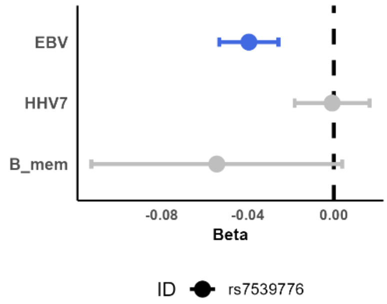

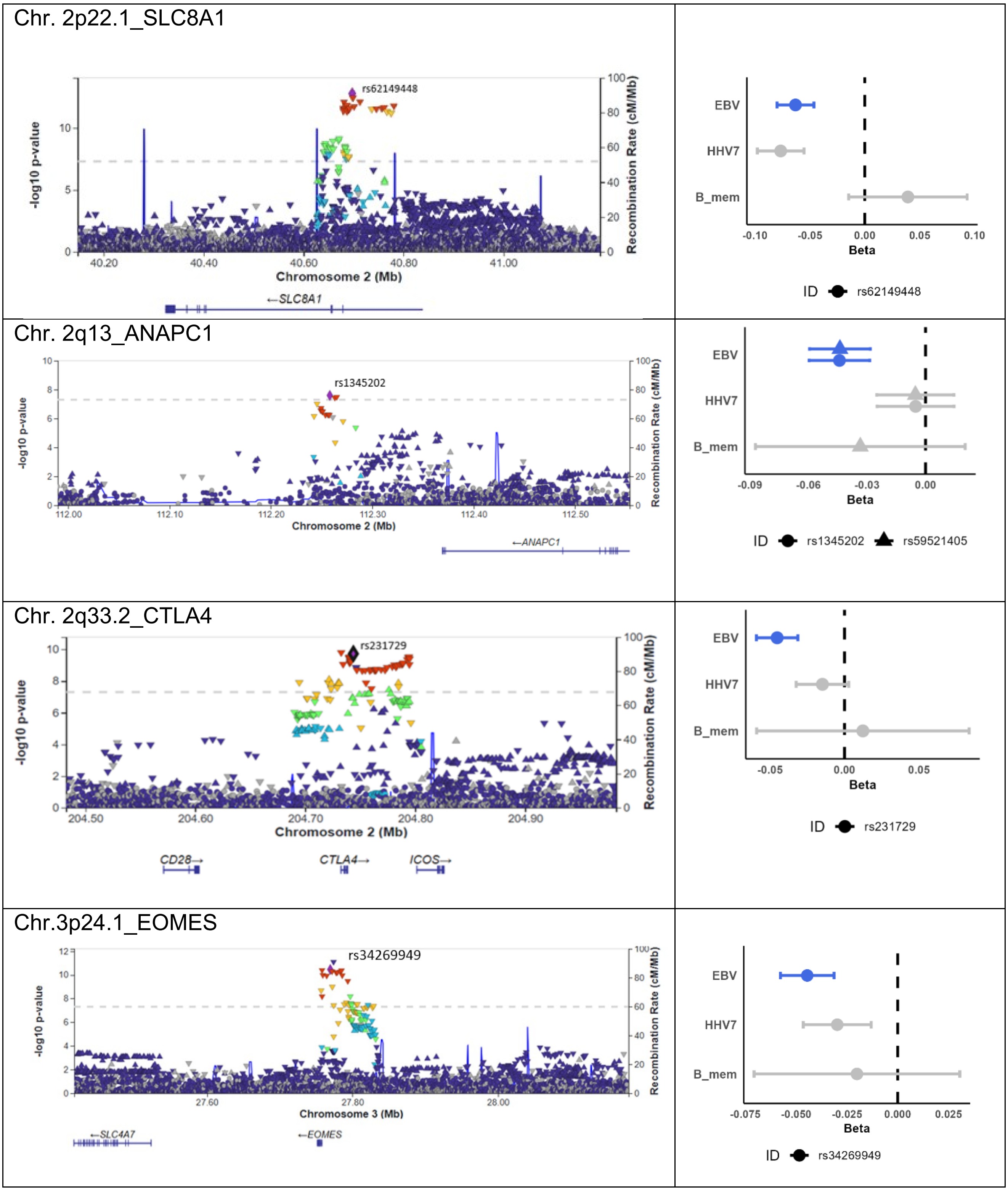

Chr. 2p22.1_SLC8A1
rs62149448
-log10 p-value
Recombination Rate (cM/Mb)
Chromosome 2 (Mb)
←SLC8A1
EBV
HHV7
B_mem
Beta
ID rs62149448
Chr. 2q13_ANAPC1
rs1345202
Chromosome 2 (Mb)
←ANAPC1
ID rs1345202 rs59521405
Chr. 2q33.2_CTLA4
rs231729
Chromosome 2 (Mb)
CD28→
CTLA4→
ICOS→
ID rs231729
Chr.3p24.1_EOMES
rs34269949
Chromosome 3 (Mb)
←SLC4A7
←EOMES
ID rs34269949

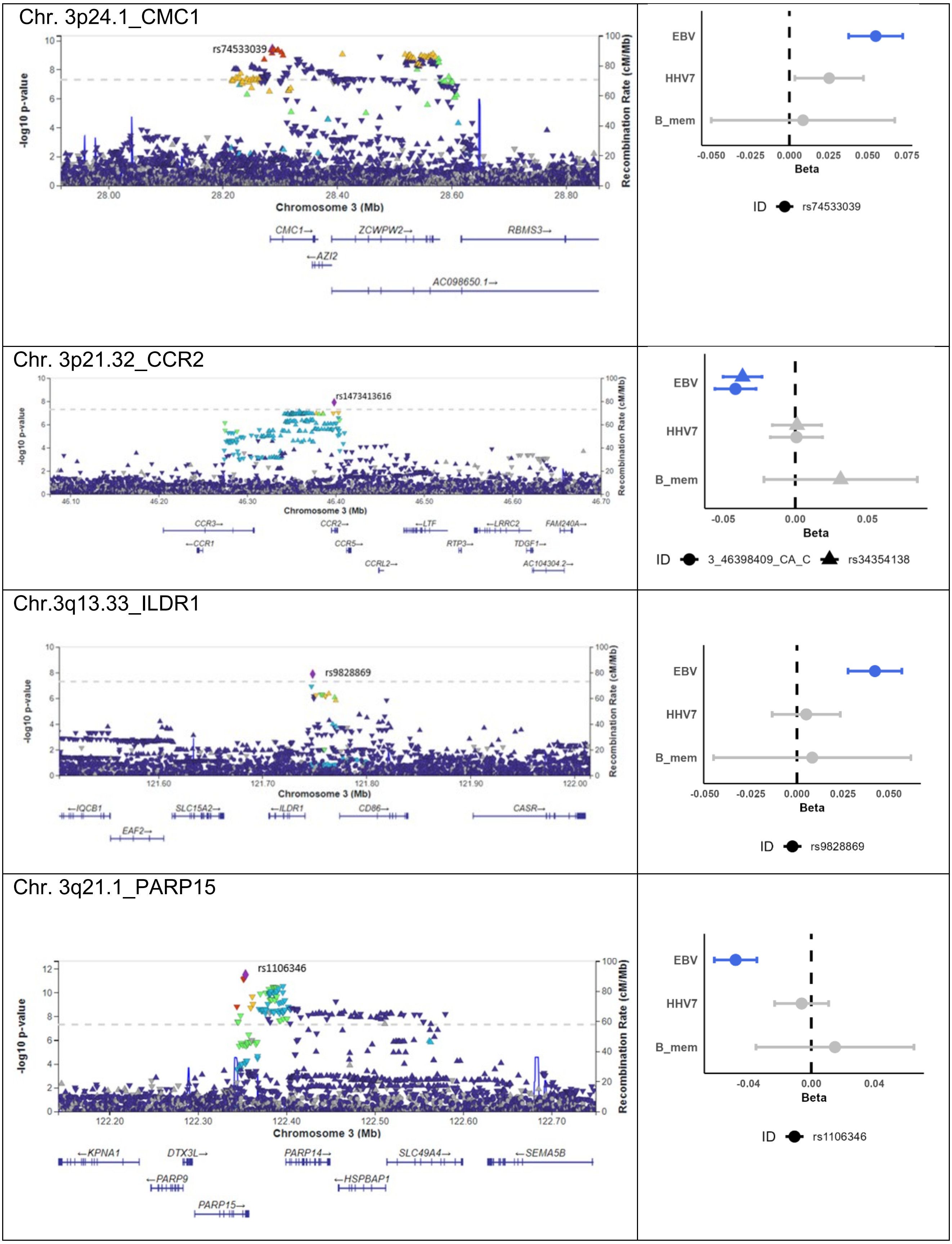

Chr. 3p24.1_CMC1
rs74533039
-log10 p-value
Recombination Rate (cM/Mb)
Chromosome 3 (Mb)
CMC1→
ZCWPW2→
RBMS3→
←AZI2
AC098650.1→
EBV
HHV7
B_mem
Beta
ID
rs74533039
Chr. 3p21.32_CCR2
rs1473413616
-log10 p-value
Recombination Rate (cM/Mb)
Chromosome 3 (Mb)
CCR3→
CCR2→
←LTF
←LRRC2
FAM240A→
←CCR1
CCR5→
RTP3→
TDGF1→
CCRL2→
AC104304.2→
EBV
HHV7
B_mem
Beta
ID
3_46398409_CA_C
rs34354138
Chr.3q13.33_ILDR1
rs9828869
-log10 p-value
Recombination Rate (cM/Mb)
Chromosome 3 (Mb)
←IQCB1
SLC15A2→
←ILDR1
CD86→
CASR→
EAF2→
EBV
HHV7
B_mem
Beta
ID
rs9828869
Chr. 3q21.1_PARP15
rs1106346
-log10 p-value
Recombination Rate (cM/Mb)
Chromosome 3 (Mb)
←KPNA1
DTX3L→
PARP14→
SLC49A4→
←SEMA5B
←PARP9
←HSPBAP1
PARP15→
EBV
HHV7
B_mem
Beta
ID
rs1106346

Chr. 3q28_LPP

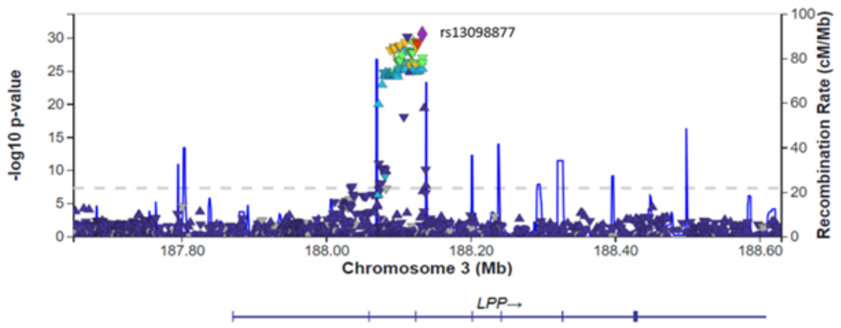

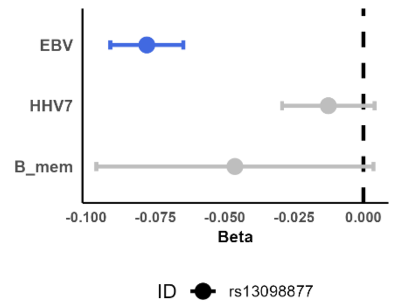

Chr. 3q28_TP63

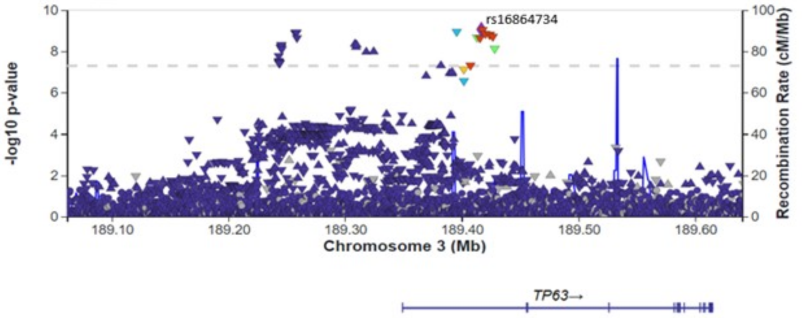

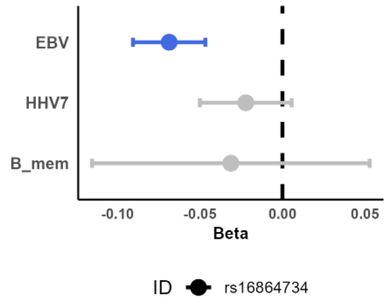

Chr. 5q15_ERAP2

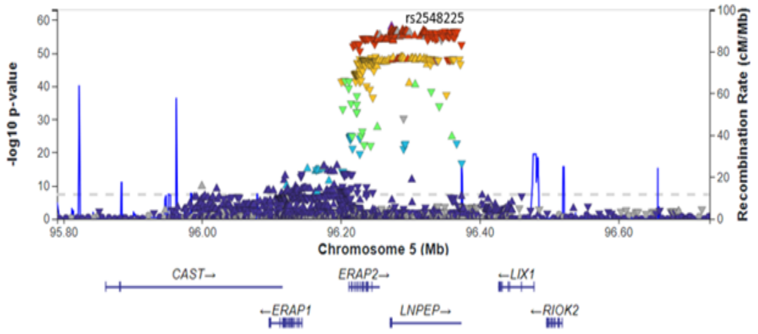

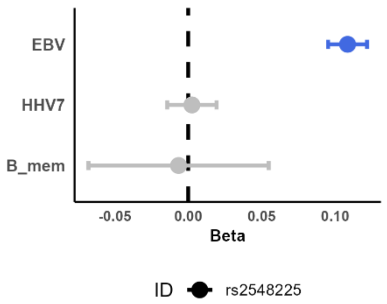

Chr. 5q31.1_IRF1

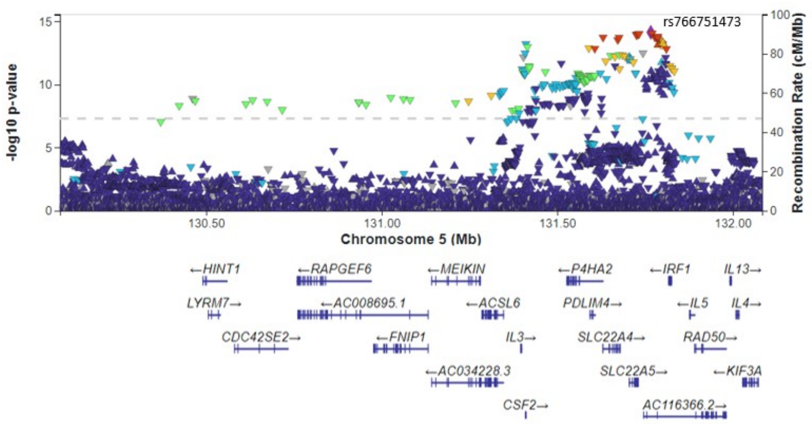

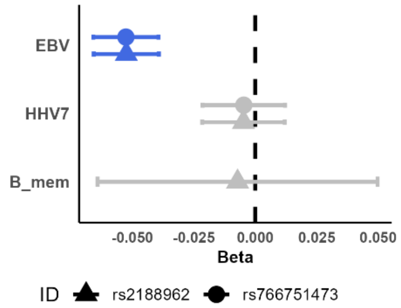

Chr. 7p12.2_SPATA48

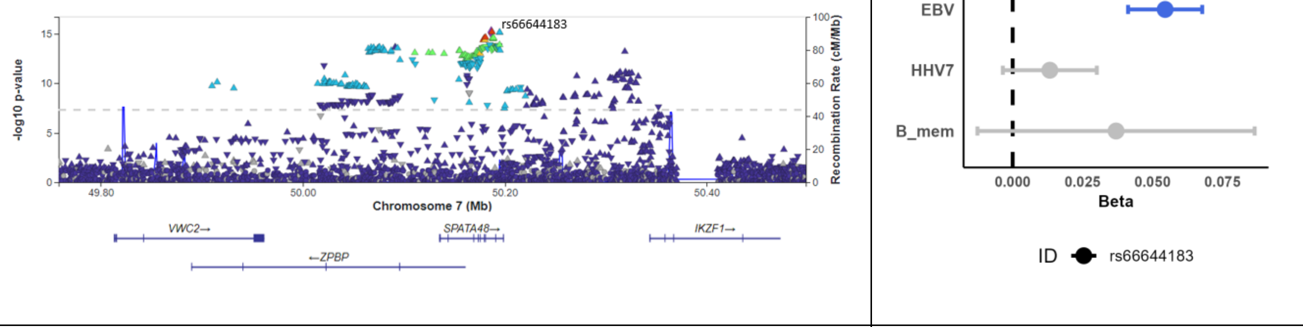

Chr. 12q24.12_SH2B3

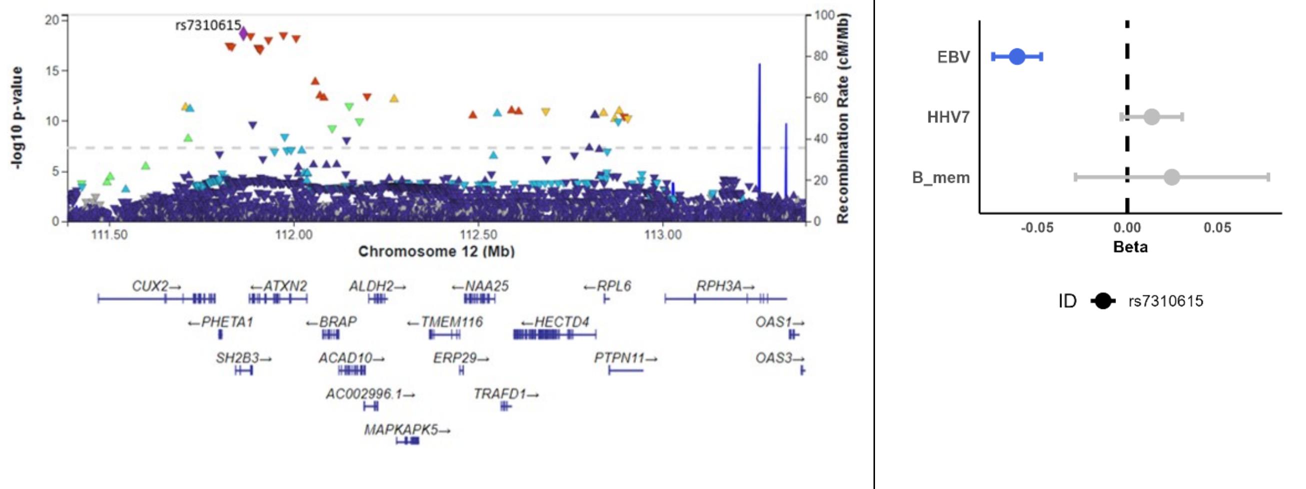

Chr. 13q14.11_FOXO1

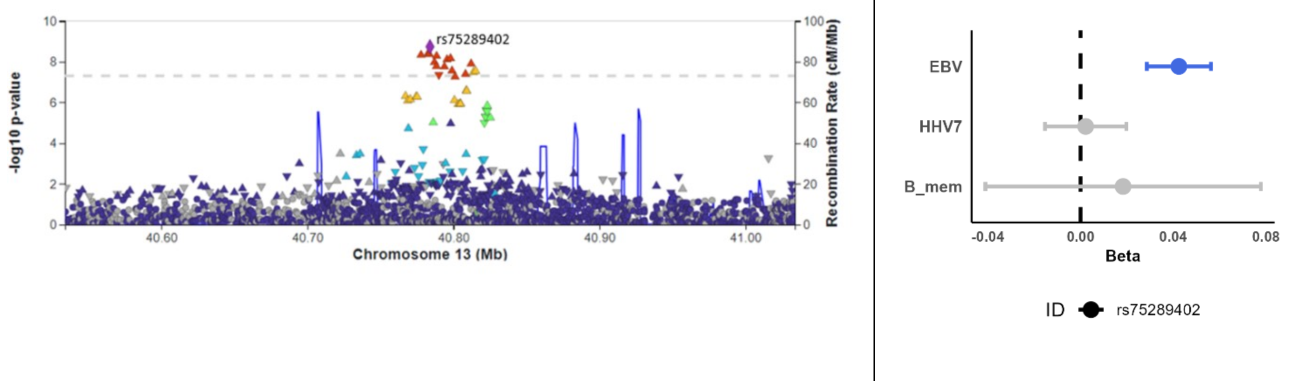

Chr. 13q32.3_UBAC2

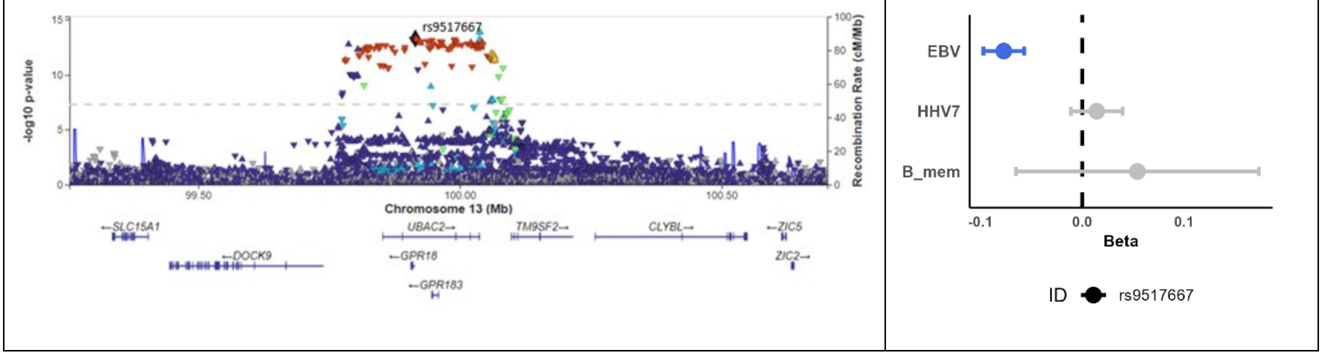

Chr. 13q33.3_TNFSF13B

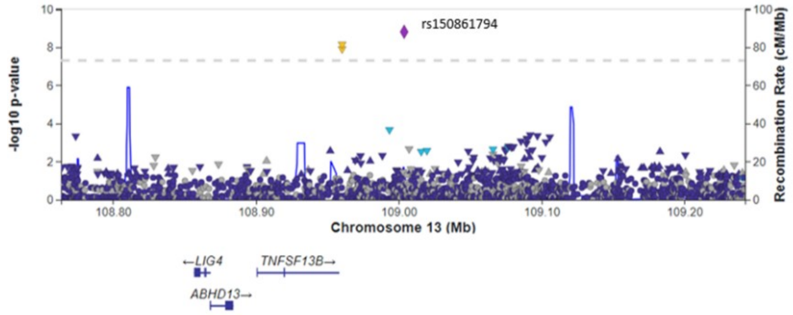

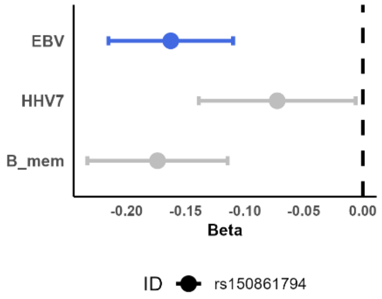

Chr. 15q15.1_CHP1

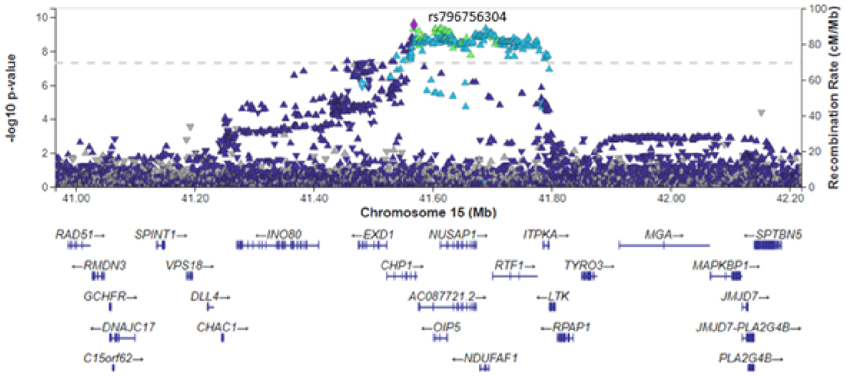

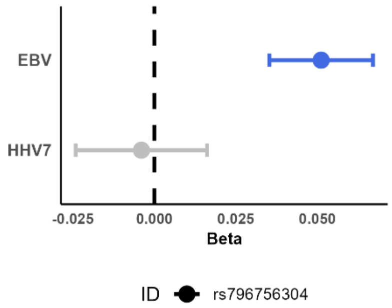

Chr. 17p11.2_TNFRSF13B

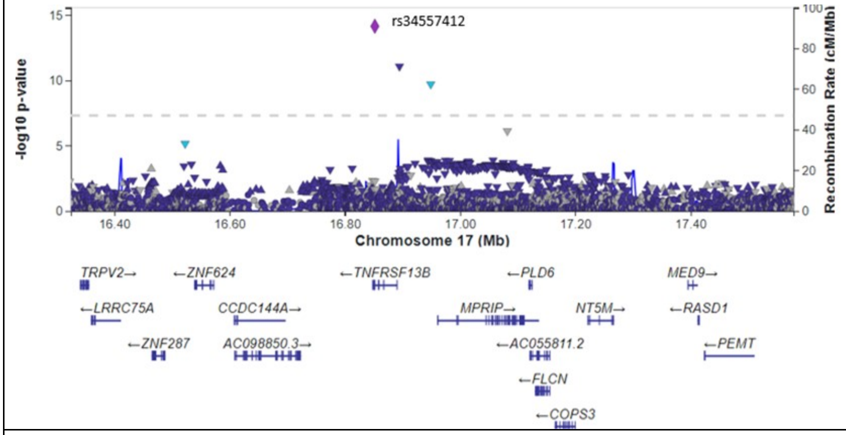

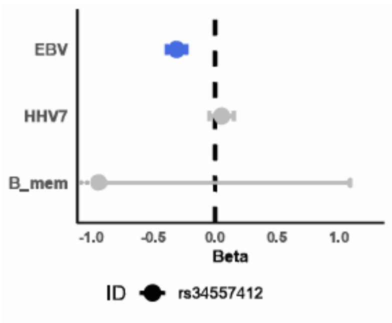

Chr. 17q11.1_KSR1

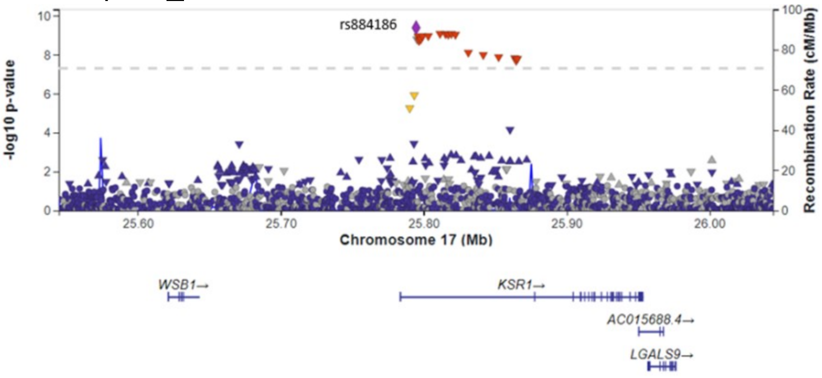

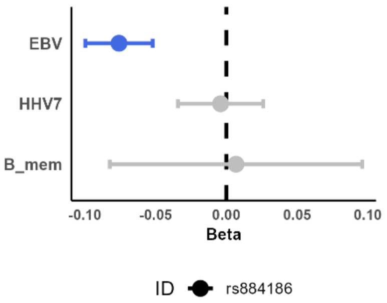

Chr. 17q12_GRB7 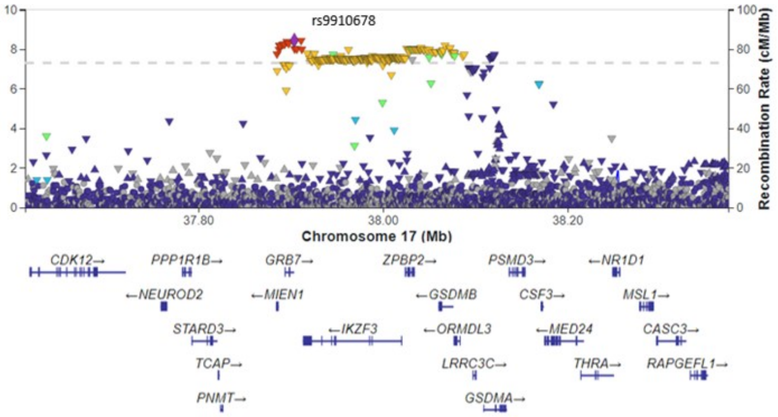	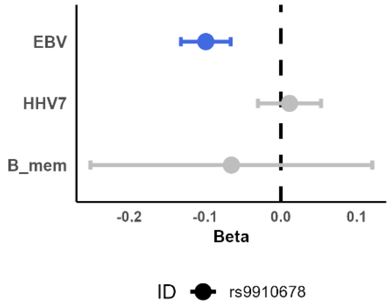
Chr. 19p13.3_CD70 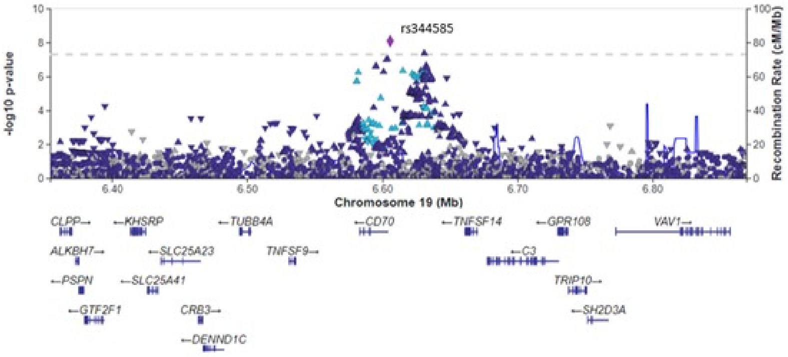	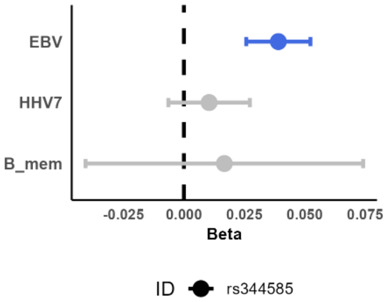
Chr. 19q13.32_BCL3 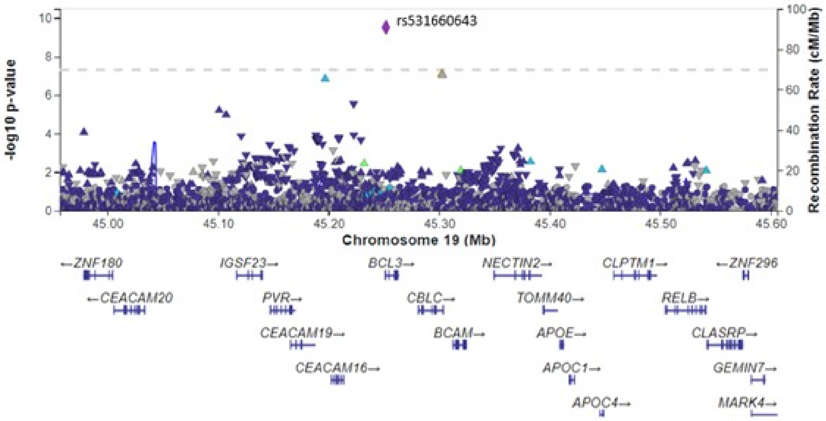	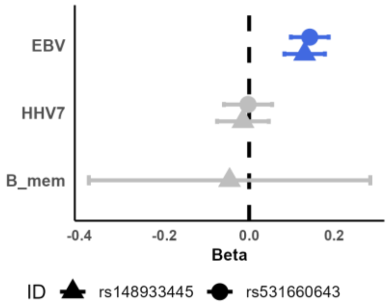

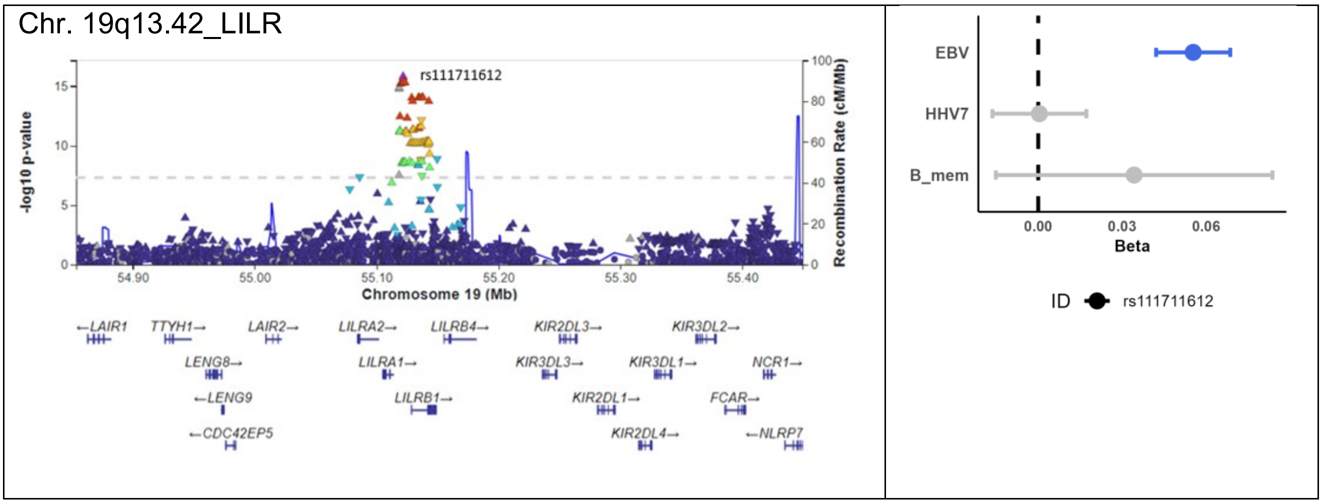

Supplementary Figure 9: 27 loci outside the MHC region were associated at genome-wide significance with EBVread+ in UKBiobank. For each locus, regional association plots (RAPs) were generated with Locus Zoom and are shown in the left column, based on the GWAS summary statistics from the main EBVread+ in UKB. The lead variant, defined as variant with lowest P-value, is diamond shaped, labeled and serves as the reference for Linkage Disequilibrium (color coded) to the remaining variants at the locus. Gene symbols and exon/intron structure are given in blue, coordinates are GRCh37-based. In the right column, Forest plots provide information on the association statistics for the lead SNP of EBVread+, and for HHV7 and memory B cells (see Methods). If the lead SNP could not be found, a proxy SNP is additionally depicted. Symbols correspond to point estimates of effect size (beta), error bars to 95% confidence intervals.

Supplementary Note 15: scDRS analyses

We downloaded single-cell RNA-seq (scRNA-seq) data from peripheral mononuclear blood cells (PBMCs) from the 1M-scBloodNL project, published by the sc-eQTLGen consortium[50]. QC-ed raw gene expression counts of the samples processed with 10x Genomics v3 as well as the corresponding genes, barcodes, and cell type annotations were downloaded on January 30, 2025. The dataset contained two levels of cell type annotations (level 1: 10 cell types; level 2: 26 cell types), of which we focused on the broader annotation level for the main analyses. Using the Seurat package v5.2.1 in R v4.3.2, the scRNA-seq dataset was filtered for the subset of untreated cells and for level 1 cell type clusters containing more than 100 cells, leading to the exclusion of cells annotated as "unknown" (77 cells) or "plasma B" (80 cells). 37,033 cells annotated to 8 cell types remained for the scDRS analysis. The dataset was written to a H5AD file for input to scDRS using the SeuratDisk package v0.0.0.9021. The top 1000 EBVread+ MAGMA genes and their z-scores as weights were obtained using the scdrs munge-gs command. The scdrs compute_score command was used with default parameters on the prepared scRNA-seq dataset and MAGMA gene set to calculate scDRS.

References Supplementary Information

98. Neitzel, H. A routine method for the establishment of permanent growing lymphoblastoid cell lines. *Hum. Genet.* **73**, 320–326 (1986).
99. Muller, C. J. & MacLehose, R. F. Estimating predicted probabilities from logistic regression: different methods correspond to different target populations. *Int. J. Epidemiol.* **43**, 962–970 (2014).
100. Yamashita, M. *et al.* A variant in human AIOLOS impairs adaptive immunity by interfering with IKAROS. *Nat. Immunol.* **22**, 893–903 (2021).
101. Lorenzini, T. *et al.* Characterization of the clinical and immunologic phenotype and management of 157 individuals with 56 distinct heterozygous NFKB1 mutations. *J. Allergy Clin. Immunol.* **146**, 901–911 (2020).
102. Schwab, C. *et al.* Phenotype, penetrance, and treatment of 133 cytotoxic T-lymphocyte antigen 4-insufficient subjects. *J. Allergy Clin. Immunol.* **142**, 1932–1946 (2018).
103. Schubert, D. *et al.* Autosomal dominant immune dysregulation syndrome in humans with CTLA4 mutations. *Nat. Med.* **20**, 1410–1416 (2014).
104. Kuehn, H. S. *et al.* Immune dysregulation in human subjects with heterozygous germline mutations in CTLA4. *Science* **345**, 1623–1627 (2014).
105. Ghosh, S. *et al.* Extended clinical and immunological phenotype and transplant outcome in CD27 and CD70 deficiency. *Blood* **136**, 2638–2655 (2020).
106. Kermasson, L. *et al.* Inherited human Apollo deficiency causes severe bone marrow failure and developmental defects. *Blood* **139**, 2427–2440 (2022).
107. Bandea, C. I., Yang, N. Q. & Wu, G. J. Screening mutants by a modified Sanger's dideoxy sequencing method in 96-well microtitre trays. *BioTechniques* **7**, 142–143 (1989).
108. Boutboul, D. *et al.* Dominant-negative IKZF1 mutations cause a T, B, and myeloid cell combined immunodeficiency. *J. Clin. Invest.* **128**, 3071–3087 (2018).
109. Hoshino, A. *et al.* Gain-of-function IKZF1 variants in humans cause immune dysregulation associated with abnormal T/B cell late differentiation. *Sci. Immunol.* **7**, eabi7160 (2022).
110. Rosain, J. *et al.* Human IRF1 governs macrophagic IFN-γ immunity to mycobacteria. *Cell* **186**, 621-645.e33 (2023).
111. Neehus, A.-L. *et al.* Human inherited CCR2 deficiency underlies progressive polycystic lung disease. *Cell* **187**, 390-408.e23 (2024).
112. Grimbacher, B. *et al.* Homozygous loss of ICOS is associated with adult-onset common variable immunodeficiency. *Nat. Immunol.* **4**, 261–268 (2003).
113. Chang, C. C. *et al.* Second-generation PLINK: rising to the challenge of larger and richer datasets. *GigaScience* **4**, 7 (2015).
114. Abraham, G., Qiu, Y. & Inouye, M. FlashPCA2: principal component analysis of Biobank-scale genotype datasets. *Bioinforma. Oxf. Engl.* **33**, 2776–2778 (2017).
115. Luo, Y. *et al.* A high-resolution HLA reference panel capturing global population diversity enables multi-ancestry fine-mapping in HIV host response. *Nat. Genet.* **53**, 1504–1516 (2021).
116. Lee, H. & Kingsford, C. Kourami: graph-guided assembly for novel human leukocyte antigen allele discovery. *Genome Biol.* **19**, 16 (2018).

117. Jia, X. *et al.* Imputing amino acid polymorphisms in human leukocyte antigens. *PloS One* **8**, e64683 (2013).
118. Kimura, H. *et al.* Quantitative analysis of Epstein-Barr virus load by using a real-time PCR assay. *J. Clin. Microbiol.* **37**, 132–136 (1999).
119. Conte, J., Potoczniak, M. J. & Tobe, S. S. Using synthetic oligonucleotides as standards in probe-based qPCR. *BioTechniques* **64**, 177–179 (2018).
120. Sasa, N. *et al.* Blood DNA virome associates with autoimmune diseases and COVID-19. *Nat. Genet.* **57**, 65–79 (2025).
