# Supplementary material for: Host control of persistent Epstein–Barr virus infection
Source: Nature. 2026 Feb 19;653(8114):444–54. doi: 10.1038/s41586-026-10274-4 (PMC13171444; doi:10.1038/s41586-026-10274-4)
Supplement: Supplementary file 2 — Reporting Summary [file 41586_2026_10274_MOESM2_ESM.pdf]

nature portfolio

Corresponding author(s): Dr. Kerstin Ludwig

Last updated by author(s): Feb 6th, 2026

Reporting Summary

Nature Portfolio wishes to improve the reproducibility of the work that we publish. This form provides structure for consistency and transparency in reporting. For further information on Nature Portfolio policies, see our Editorial Policies and the Editorial Policy Checklist.

Statistics

For all statistical analyses, confirm that the following items are present in the figure legend, table legend, main text, or Methods section.

n/a	Confirmed	
☐	☒	The exact sample size (*n*) for each experimental group/condition, given as a discrete number and unit of measurement
☐	☒	A statement on whether measurements were taken from distinct samples or whether the same sample was measured repeatedly
☐	☒	The statistical test(s) used AND whether they are one- or two-sided<br>*Only common tests should be described solely by name; describe more complex techniques in the Methods section.*
☐	☒	A description of all covariates tested
☐	☒	A description of any assumptions or corrections, such as tests of normality and adjustment for multiple comparisons
☐	☒	A full description of the statistical parameters including central tendency (e.g. means) or other basic estimates (e.g. regression coefficient) AND variation (e.g. standard deviation) or associated estimates of uncertainty (e.g. confidence intervals)
☐	☒	For null hypothesis testing, the test statistic (e.g. *F*, *t*, *r*) with confidence intervals, effect sizes, degrees of freedom and *P* value noted<br>*Give P values as exact values whenever suitable.*
☒	☐	For Bayesian analysis, information on the choice of priors and Markov chain Monte Carlo settings
☒	☐	For hierarchical and complex designs, identification of the appropriate level for tests and full reporting of outcomes
☐	☒	Estimates of effect sizes (e.g. Cohen's *d*, Pearson's *r*), indicating how they were calculated

Our web collection on statistics for biologists contains articles on many of the points above.

Software and code

Policy information about availability of computer code

Data collection	We analysed existing genome sequencing data and phenotypic information from the two large biobanks UKBiobank (UKB) and All of Us (AoU). Reads mapping to the EBV or HHV7 genome were extracted from GS-derived CRAM files within the frameworks snakemake (v.7.32.4; UKB) or nextflow (v25.04; AoU). In particular, read extraction and filtering was performed using samtools (UKB: v1.20, AoU: v1.22), alignment of reads to the HHV7 genome with bwa-mem2 (v2.2.1). Viral reads were visualized with IGV (v2.12.3).<br>Common variants for association analyses were retrieved from data field 22828 (imputed genotypes, bgen format) in UKB, and from GS-based variant call plink2 files for AoU. Rare variants (UKB, exome sequencing data) were retrieved from data field 23158. HLA-alleles were retrieved from field 22182 in UKB, or imputed based on genotype data (plink1 file format) using HLA-TAPAS for AoU (https://github.com/immunogenomics/HLA-TAPAS). Phenome-wide analyses were conducted in AoU based on SNOMED-IDs as provided in the AoU database.<br>Code to extract and quantify EBV-reads is archived in the repository EBVread-extraction (https://github.com/Ax-Sch/EBVread-extraction), and analysis code can be found within the repository EBVread_data_analysis (https://github.com/Ax-Sch/EBVread-data-analysis). Archived versions of the repositories are also available via zenodo (10.5281/zenodo.18417294).

Data analysis	Data analysis was performed within the frameworks R (v4.3.2 and higher, i.e. UKB: v4.4.0, AoU: v4.5.0; tidyverse v2.0.0) and python (UKB: v3.9.16, AoU: v3.10.16). Variant level genetic data was analyzed and handled with plink (UKB: v1.90b7.4; and v1.90b6.21 in GRS-scoring; AoU: v1.90b6.22 and v1.9.0-b.7.7 in GRS-scoring), plink2 (v2.0.0-a.6), bcftools (UKB: v1.20, AoU: v1.12) and FlashPCA (v2.0). Association analysis was performed using Regenie v3.24 (UKB) or v2.0.2 (AoU). Covariates were analyzed using R libraries 'splines' (v4.4) and 'MASS' (v7.3-6). Typing of HLA-alleles was performed using kourami (v0.9.6). Downstream analyses and visualizations were performed using FUMA (v1.6.3), MAGMA (v1.08), OpenTargets (v22.10, v25.3), Ensembl VEP (v113.0), coloc (v.5.2.3), scDRS (v1.0.3), seurat (v5.2.1), PRS-CS (v1.0.0), PheTK (v0.1.47), PheCodes (v1.2), ieugwasr (v1.0.3), TwoSampleMR (v0.6.15), MR-PRESSO (https://github.com/rondolab/MR-PRESSO), MR_RAPS (arXiv:1801.09652), LDSR (v1.0.1), SuSie (v0.15.4), Seurat Disk (v0.0.0.9021). Processing of RNAseq-data: STAR (v2.5.3a) and RSEM (v.1.3.0). See "Data collection" for custom code availability.

For manuscripts utilizing custom algorithms or software that are central to the research but not yet described in published literature, software must be made available to editors and reviewers. We strongly encourage code deposition in a community repository (e.g. GitHub). See the Nature Portfolio guidelines for submitting code & software for further information.

Data

Policy information about availability of data

All manuscripts must include a data availability statement. This statement should provide the following information, where applicable:

- Accession codes, unique identifiers, or web links for publicly available datasets
- A description of any restrictions on data availability
- For clinical datasets or third party data, please ensure that the statement adheres to our policy

All genetic and phenotype data from the biobanks are available upon application and approved data access from the UK Biobank study and AllofUs projects. All interested readers will be able to access the data in the same manner that the authors did, including usage of the UKB Research Analysis Platform and AoU workbench environments for the analysis of de-identified individual-level data. GWAS summary statistics are available through the GWAS catalog (GCST GCST90809298–GCST90809306). All additional data are either provided in Supplementary Tables or through Zenodo (10.5281/zenodo.18417294), including the custom code repository as .zip files (EBVread-data-analysis-main_1.0.zip; EBVread-extraction-main_1.0.zip). Complementary data used for secondary analyses were obtained from: OneK1K (https://onek1k.org/), eQTLgen 1M-scBloodNL (https://www.eqtlgen.org/sc/datasets/1m-scbloodnl-dataset.html), GTEx (https://www.gtexportal.org/home/), OpenTargets (https://platform.opentargets.org/), IUIS (https://iuis.org/committees/iei/), GWAS Catalogue (https://www.ebi.ac.uk/gwas/), the International Multiple Sclerosis Genomics consortium (https://imsgc.net/). Data access for the two validation cohorts is described in their respective original articles (references PMIDs: 35923707 (validation cohort-1), 39317738 (validation cohort-2)).

Research involving human participants, their data, or biological material

Policy information about studies with human participants or human data. See also policy information about sex, gender (identity/presentation), and sexual orientation and race, ethnicity and racism.

Reporting on sex and gender	Analyses were performed including males and females. Sex was used as covariate in many statistical analyses and was determined based on information reported in UKB or AoU via genetic inference.
Reporting on race, ethnicity, or other socially relevant groupings	Within the UKB dataset, individuals of European population were selected based on information given in UK Biobank field 22006, i.e. self reported 'White British' ethnicity and very similar genetic ancestry based on a principal components analysis (PCA) of the genotypes. In AoU, we used precomputed genetically predicted population backgrounds, which assigned each individual to one of six continental populations (African, Admixed American, East Asian, European, Middle Eastern, South Asian; see AoU Genomic Research Data Quality Report).
Population characteristics	We used all individuals of the UKB and AoU projects for whom blood-based genome sequencing data were available following quality control. Discovery analyses were performed in the UKB-QC-cohort (mixed ancestry, mean age of 56.5 years, 54.2% being female). In the AoU-QC cohort used for replication analyses, ancestry groups were as follows: Africans (mean age 49.3 years, 57.1% female), Admixed Americans (44.5 years, 65.0% female), East Asian (43.45 years, 63.0% female), European (55.5 years, 59.1% female), Middle Eastern (44.4 years, 52.5% female), South Asian (40.8 years, 52.8% female). Validation cohorts comprised European (validation-1: 67.3 years, 48.6% female) or EastAsian (validation-2: 60.8 years, 48.1% female) individuals.
Recruitment	No recruitment of participants was performed in this study as we used existing cohorts and datasets. More information on the recruitment for UKB and AoU can be found in previously published work. UKB: Sudlow et al., 2015 PLOS Medicine; Bycroft et al., 2018, Nature; Halldorsson et al., 2022 Nature; UKB WGS consortium, 2025, Nature; AoU: All of Us Research Program Investigators, 2019 NEJM; AoU Genomic Program, 2024, Nature. Recruitment of the two validation cohorts has been described previously (references PMIDs: 35923707 (validation cohort-1), 39317738 (validation cohort-2)).
Ethics oversight	This study used de-identified data available from UKB and AoU, which were accessed through their respective platforms. UKB has approval from the North West Multi-centre Research Ethics Committee (MREC) as a Research Tissue Bank (RTB). This approval means that researchers do not require separate ethical clearance and can operate under the RTB approval. The data collection of the AoU Research Program was conducted under centralized Institutional Review Board (IRB) approval, with informed consent obtained from participants. UKB Tier-3 data was accessed based on application-ID 135122. For the two validation cohorts, ethical approvals were obtained from the Ethics Committee of the Medical Faculty Bonn (no. 101/16; for analysis of validation cohort 1) and by the ethical committees of the affiliated institutes (Keio IRB approval 20200061, Osaka University IRB approval 734-14, University of Tsukuba IRB approval H29-294) for the JCTF.

Note that full information on the approval of the study protocol must also be provided in the manuscript.

Field-specific reporting

Please select the one below that is the best fit for your research. If you are not sure, read the appropriate sections before making your selection.

☒ Life sciences ☐ Behavioural & social sciences ☐ Ecological, evolutionary & environmental sciences

For a reference copy of the document with all sections, see nature.com/documents/nr-reporting-summary-flat.pdf

Life sciences study design

All studies must disclose on these points even when the disclosure is negative.

Sample size	We included all individuals of UKB and AoU for whom genome sequencing data from blood were available. For each analysis we maximized the number of used samples based on quality control measures, without a priori sample size calculation. The total sample size for EBV-read extraction after quality control was 486,315 individuals for UKB and 336,123 for AoU. For the validation cohorts, sample sizes were determined by the number of individuals in each study.
Data exclusions	Data were excluded during the study procedure for quality control reasons, in particular: low-quality genome sequencing data, outliers during library preparation, implausible covariates, missing data. All details are provided in the Methods section.
Replication	Whenever possible, we used one of the two biobanks for discovery, and the other one for replication. This is illustrated as Supplementary Note 1. Specifically, for the genetic data, we aimed to replicate the results of the main EBVread+ GWAS from UKB in 184,948 individuals of European ancestry from AoU. We observed nominal significance and consistent effect direction for 100 of 106 HLA alleles that could be matched across both datasets and for lead variants at 25 of the 27 non-MHC loci. Similar analyses were performed for a GRS generated in UKB, and for the non-genetic factors which were determined in AoU first and replicated in UKB. For non-genetic factors, all replication efforts between UKB and AoU were successful for phenotypes that were available and comparably assessed in both biobanks.
Randomization	This population-based study is observational, therefore randomization was not relevant for our work.<br>We extensively test and correct for potential confounders using existing phenotype and metadata in UKB and AoU, which is described in detail in the Methods section.
Blinding	Blinding was not relevant for this study as experimental group assignment was not performed.

Reporting for specific materials, systems and methods

We require information from authors about some types of materials, experimental systems and methods used in many studies. Here, indicate whether each material, system or method listed is relevant to your study. If you are not sure if a list item applies to your research, read the appropriate section before selecting a response.

Materials & experimental systems

n/a	Involved in the study	
☒	☐	Antibodies
☒	☐	Eukaryotic cell lines
☒	☐	Palaeontology and archaeology
☒	☐	Animals and other organisms
☒	☐	Clinical data
☒	☐	Dual use research of concern
☒	☐	Plants

Methods

n/a	Involved in the study	
☒	☐	ChIP-seq
☒	☐	Flow cytometry
☒	☐	MRI-based neuroimaging

Plants

Seed stocks	NA
Novel plant genotypes	NA
Authentication	NA
